# Circ_0008542 in osteoblast exosomes promotes osteoclast-induced bone resorption through m6A methylation

**DOI:** 10.1038/s41419-021-03915-1

**Published:** 2021-06-18

**Authors:** Wei Wang, Shi-Chong Qiao, Xiang-Bing Wu, Bao Sun, Jin-Gang Yang, Xing Li, Xiao Zhang, Shu-Jiao Qian, Ying-Xin Gu, Hong-Chang Lai

**Affiliations:** 1 Department of Implant Dentistry, Shanghai Ninth People’s Hospital, College of Stomatology, Shanghai Jiao Tong University School of Medicine, National Clinical Research Center for Oral Diseases, Shanghai Key Laboratory of Stomatology & Shanghai Research Institute of Stomatology, No. 639 Zhizaoju Road, Shanghai, 200011 China; 2 Department of Oral Pathology, Shanghai Ninth People’s Hospital, College of Stomatology, Shanghai Jiao Tong University School of Medicine, National Clinical Research Center for Oral Diseases, Shanghai Key Laboratory of Stomatology & Shanghai Research Institute of Stomatology, No. 639 Zhizaoju Road, Shanghai, 200011 China; 3 Department of Orthodontics, Shanghai Ninth People’s Hospital, College of Stomatology, Shanghai Jiao Tong University School of Medicine, National Clinical Research Center for Oral Diseases, Shanghai Key Laboratory of Stomatology & Shanghai Research Institute of Stomatology, No. 639 Zhizaoju Road, Shanghai, 200011 China; 4 Department of Oral Maxillofacial-Head Neck Oncology, Shanghai Ninth People’s Hospital, College of Stomatology, Shanghai Jiao Tong University School of Medicine, National Clinical Research Center for Oral Diseases, Shanghai Key Laboratory of Stomatology & Shanghai Research Institute of Stomatology, No. 639 Zhizaoju Road, Shanghai, 200011 China

**Keywords:** Epigenetics, Predictive markers

## Abstract

With an increasing aging society, China is the world’s fastest growing markets for oral implants. Compared with traditional oral implants, immediate implants cause marginal bone resorption and increase the failure rate of osseointegration, but the mechanism is still unknown. Therefore, it is important to further study mechanisms of tension stimulus on osteoblasts and osteoclasts at the early stage of osseointegration to promote rapid osseointegration around oral implants. The results showed that exosomes containing circ_0008542 from MC3T3-E1 cells with prolonged tensile stimulation promoted osteoclast differentiation and bone resorption. Circ_0008542 upregulated Tnfrsf11a (RANK) gene expression by acting as a miR-185-5p sponge. Meanwhile, the circ_0008542 1916-1992 bp segment exhibited increased m6A methylation levels. Inhibiting the RNA methyltransferase METTL3 or overexpressing the RNA demethylase ALKBH5 reversed osteoclast differentiation and bone resorption induced by circ_0008542. Injection of circ_0008542 + ALKBH5 into the tail vein of mice reversed the same effects in vivo. Site-directed mutagenesis study demonstrated that 1956 bp on circ_0008542 is the m6A functional site with the abovementioned biological functions. In conclusion, the RNA methylase METTL3 acts on the m6A functional site of 1956 bp in circ_0008542, promoting competitive binding of miRNA-185-5p by circ_0008542, and leading to an increase in the target gene RANK and the initiation of osteoclast bone absorption. In contrast, the RNA demethylase ALKBH5 inhibits the binding of circ_0008542 with miRNA-185-5p to correct the bone resorption process. The potential value of this study provides methods to enhance the resistance of immediate implants through use of exosomes releasing ALKBH5.

## Introduction

Maximizing osseointegration is the key factor in successful implant placement. At the interface where osseointegration occurs, osteoclast-induced bone resorption and osteoblast-induced bone formation are in a dynamic balance. However, osteoclast differentiation and bone resorptions enhance, and disrupt the balance of bone remodeling during some pathological conditions [1, 2]. It is well known that immediate implantation of a single tooth or partial multitooth loss will increase the difficulty of stress load control and increase the possibility of osseointegration failure. Previous studies have found that immediate loading by implants can easily lead to bone resorption at the edge of the implant neck [3]. This indicates that the stress concentration of the implant neck has a potential relationship with bone resorption at the implant neck edge.

Exosomes are widely distributed, contain proteins and nucleic acids and are involved in various physiological and pathological processes [4]. Since communication between osteoblasts and osteoclasts is crucial for regulating environmental stability in bone tissues, exosomes can be transported to adjacent or distant cells to send a series of signals affecting environmental stability in bone tissues and regulate bone growth and homeostasis [5]. The exosome levels released by cells are significantly increased under pathological conditions, and the contents are significantly different from those in physiological conditions. This demonstrates that the donor cells exhibit precise targeted regulation of exosomal contents and reflects the significance of differential molecules in the process of disease formation. In recent years, in addition to the proven classical signaling pathways RANKL-RANK-OPG and Ephrin-Eph, interactions between osteoblasts and osteoclasts regulated by exosomes have attracted increasing attention, revealing many mechanisms related to bone metabolism and bone diseases [6–9].

Exosomes contain a variety of noncoding RNAs which become a new target for clinical diagnosis and prognostic assessment of diseases [10, 11]. CircRNA is a noncoding RNA with a closed circular structure and is not easily degraded by RNA enzymes [12, 13]. Current research on circRNA has primarily focused on regulatory cell transcription, translation, coding proteins, and molecular sponge activity with miRNAs [14–16]. As circRNA plays an important role in a variety of diseases, it is of great research value [17, 18]. In the skeletal system, current research has confirmed that circRNAs participate in the regulation of bone remodeling through the miRNA-mRNA axis in the form of molecular sponges [19–21].

M6A methylation is the most common form of RNA methylation, and as an epigenetic modification, it participates in and regulates many important functions of RNA [22]. M6A methylation is a dynamic and reversible process. The METTL3/METTL14 methyltransferase complex and the WTAP cofactor are primarily involved in methylation of m6A, and the process of demethylation is primarily completed by the methylase FTO and ALKBH5 [23–27]. At present, most studies have focused the role of m6A modification on mRNA in posttranscriptional regulation; M6A modification on pri-miRNA promotes the generation of mature miRNA after transcription; M6A modification on circRNA also promotes circRNA translation [28–30]. Recent studies have confirmed that m6A methylation regulates the molecular sponge effect between lincRNA1281 and the let-7 miRNA family, thus affecting the normal differentiation of mouse embryonic stem cells. This RNA–RNA interaction is m6A methylation-dependent [31]. Another study performed a motif search among m6A regions of multiple cell types. Over two-thirds of identified RRACH motifs were reversely complementary to the seed sequences of one or more miRNAs, indicating that the m6A peak regions might be targeted by miRNAs [32]. However, there are currently no reports of m6A modification regulating the binding of circRNA or miRNA.

Based on the above theories, this study examined the molecular mechanism of the interaction between osteoblasts and osteoclasts in the osseointegration microenvironment. After improper tension stimulation, abnormal molecular signals within osteoblast exosomes promoted osteoclast-induced bone resorption, leading to osseointegration destruction around the implant.

## Results

### Characterization of circ_0008542

**Fig. 1:**
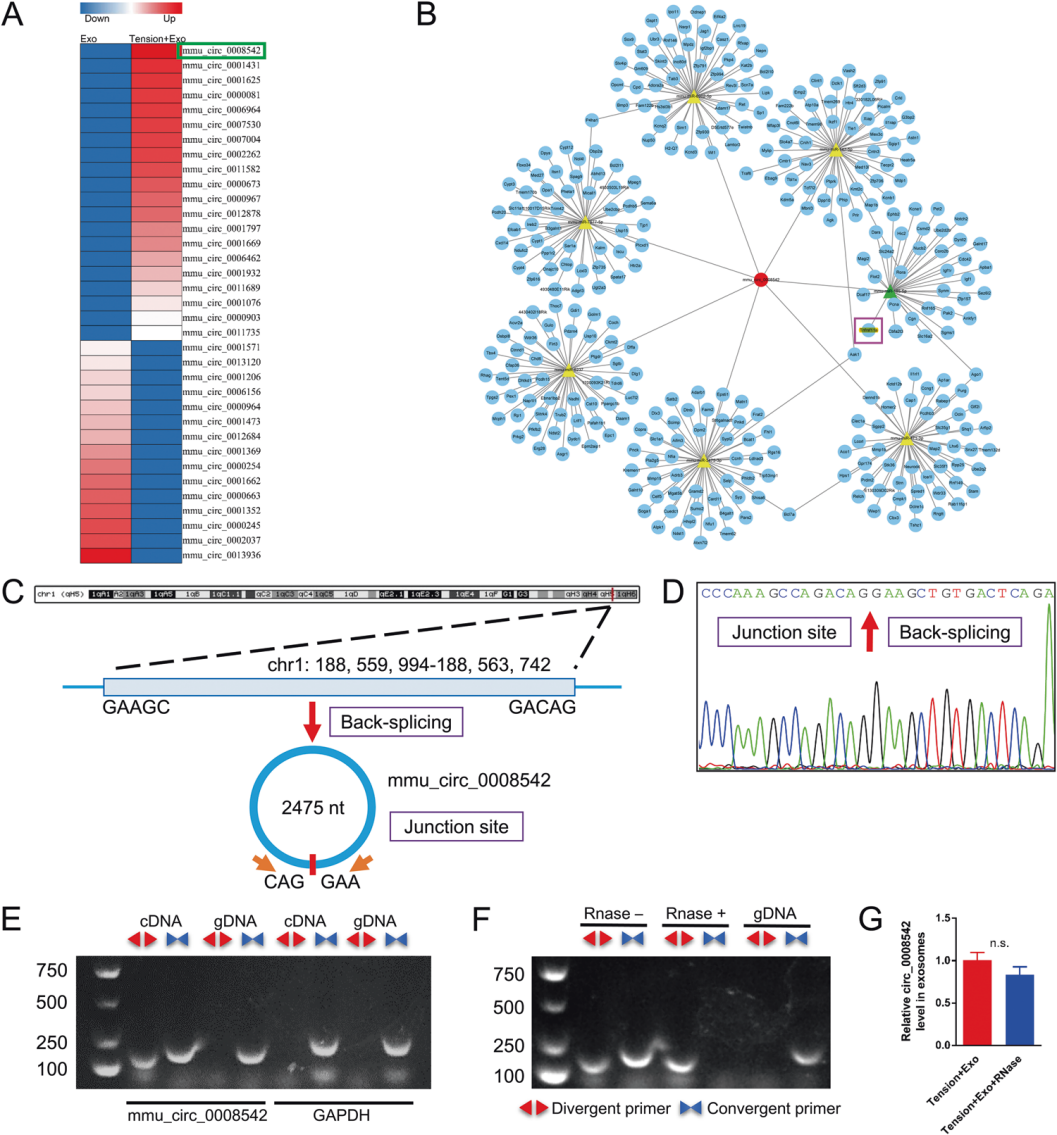
Characterization of circ_0008542. **A** A heat map of different circRNAs between the two groups with or without tension stimulation. **B** Bioinformatics prediction of the circ_0008542/miRNA-185-5p-RANK axis. **C** Schematic diagram showing the genomic location and back-splicing pattern of circ_0008542. **D** Sanger sequencing of circ_0008542. The arrow shows the “head-to-tail” splicing site. **E** Divergent primers detected circ_0008542 from cDNA by PCR and agarose gel electrophoresis, rather than from gDNA. **F** Circ_0008542 from cDNA was amplified with divergent primers, even after treatment with RNase R digestion, and the opposite results were observed for gDNA. **G** Relative expression level of circ_0008542 in exosomes between two groups with or without RNase R digestion. Data were representative of three independent experiments expressed as the mean ± SD (*p < 0.05).

Exosomes were separately collected from MC3T3-E1 cell supernatant with or without tension stimulation (Flexcell culture with 20% amplitude/1 Hz/24 h). Total RNA extracted from the abovementioned two groups was subjected to high-throughput sequencing. We observed different circRNAs between the two groups as shown in the heat map (Fig. 1A and Table S1). Circ_0008542 was highly expressed in exosomes with tension stimulation. Bioinformatics prediction results showed that circ_0008542 targets and binds to the miRNA-185-5p-RANK axis (Fig. 1B). Circ_0008542 is derived from the host gene Rrp15. It is located on chromosome 1: 188, 559, 994-188, 563,742 (2475 nt), and the genomic structure suggests that circ_0008542 consists of three exons and one intron from the Rrp15 gene locus (Fig. 1C). Circ_0008542 was demonstrated to form head-to-tail splicing and matched the sequence reported for circ_0008542 in circRase, as obtained from Sanger sequencing (Fig. 1D and Table S2). In addition, divergent and convergent primers were designed for circ_0008542, and complementary DNA (cDNA) and genomic DNA (gDNA) were used as templates. Results showed that a single and distinct product of expected size was amplified using circ_0008542 divergent primers (a 127 bp fragment) from only cDNA, while there was no amplification product from gDNA (Fig. 1E). Ribonuclease R (RNase R) digestion results showed that circ_0008542 exhibited a stable feature after RNase R treatment (Fig. 1F, G).

### Exosomes from MC3T3-E1 cells regulate osteoclast differentiation and bone resorption

**Fig. 2:**
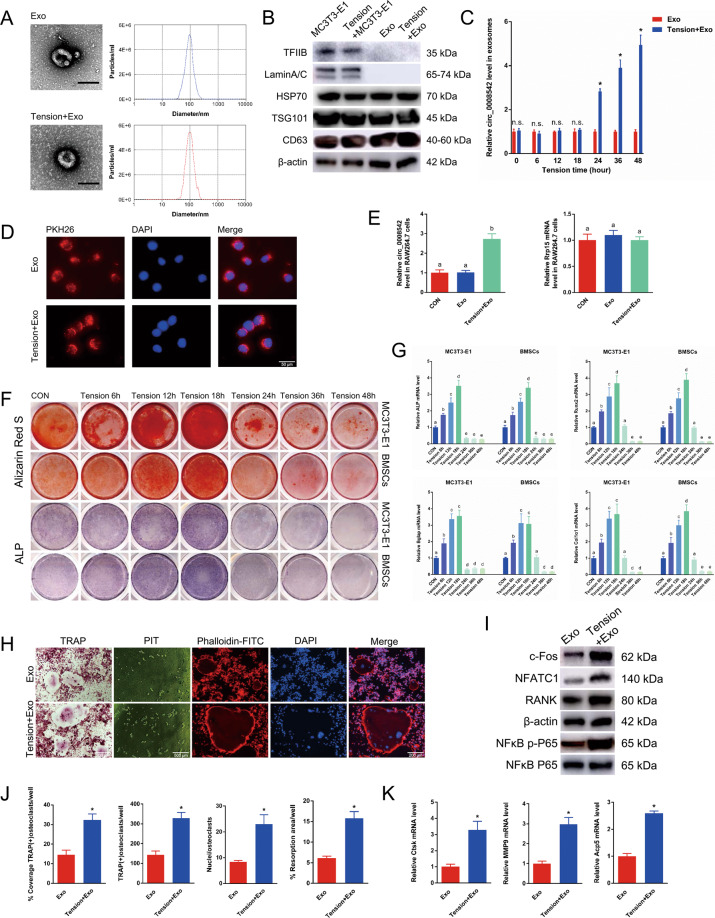
The effects of exosomes in vitro. **A** Electron microscopy images of exosomes. Scale bar, 100 nm. Size distribution of exosomes secreted by MC3T3-1 cells with or without tension stimulation. **B** Protein levels of TFIIB, Lamin A/C, HSP70, TSG101, and CD63 in MC3T3-E1 cell lysates or exosomes secreted by MC3T3-E1 cells with or without tension stimulation analyzed by western blot. **C** Relative expression level of circ_0008542 in exosomes at different tension stimulation times. **D** Immunofluorescence images of exosomes from MC3T3-E1 cells to RAW264.7 cells. Exosomes were labeled with PKH26. **E** Relative expression level of circ_0008542 or Rrp15 in RAW 264.7 cells after addition of different exosomes. **F** After different tension stimulation times, Alizarin Red S or ALP staining was applied to MC3T3-E1 cells and BMSCs after 21 days or 7 days of osteogenic induction. **G** Relative expression levels of ALP, Runx2, Bglap, and Col1α1 in MC3T3-E1 cells and BMSCs at different tension stimulation times. **H** TRAP staining, pit formation assay, and F-actin band staining were applied to detect osteoclast differentiation and bone resorption ability between the two groups with or without tension stimulation exosomes. **I** Protein levels of c-fos, NFATc1, RANK, and NFκB p-P65 in RAW 264.7 cell lysates between the two groups with or without tension stimulation exosomes were analyzed by western blot. **J** Histograms of coverage rate, number and nuclei of TRAP-positive osteoclasts, and bone resorption area rate between the two groups. **K** Relative expression levels of Ctsk, MMP9, and TRAP in RAW 264.7 cells after adding different exosomes. Data were representative of three independent experiments expressed as the mean ± SD (*p < 0.05). Different letters (a, b, c, d, and e) indicate significant differences among multiple groups (*p* < 0.05).

Representative exosomes were observed under transmission electron microscopy. The size distribution of exosomes indicated that the diameter of particles was mostly concentrated at 100 nm (Fig. 2A). Regardless of the presence or absence of additional tension stimulation, cytomembrane characteristic proteins of HSP70, TSG101, and CD63 were positive in MC3T3-E1 cell lysates and exosomes; cell nucleus characteristic proteins of TFIIB and Lamin A/C were positive in MC3T3-E1 cell lysates but negative in exosomes (Fig. 2B). Expression levels of circ_0008542 in exosomes increased with extened tension time as shown from the RT-qPCR results (Fig. 2C). Next, the immunofluorescence assay demonstrated that exosomes marked with PKH26 could be absorbed by osteoclast precursor cells (Fig. 2D). After adding exosomes from the tension stimulation group, expression levels of circ_0008542 increased in RAW 264.7 cells in the RT-qPCR results. Regardless of whether exosomes were from the groups with or without tension stimulation, there was no change in expression of Rrp15, the host gene of circ_0008542, in RAW264.7 cells, indicating that the increased circ_0008542 was derived from the exosomes (Fig. 2E). Alizarin red S (ARS) and alkaline phosphatase (ALP) staining were applied to detect osteoblast bone formation ability after different tension stimulation times. Short-term tension stimulation (less than 18 h) promoted osteoblast bone formation, but long-term tension stimulation (more than 24 h) had the opposite effect in both MC3T3-E1 cells and bone marrow stromal cells (BMSCs) (Fig. 2F). In addition, expression levels of the bone formation marker genes ALP, Runx2, Bglap, and Col1α1 in MC3T3-E1 cells and BMSCs showed the same tendency based on the RT-qPCR results (Fig. 2G). We evaluated the direct effects of exosomes on RANKL-induced osteoclast formation. From the results of tartrate-resistant acid phosphatase (TRAP) and F-actin band staining, an increasing number of osteoclasts with TRAP-positive large cell bodies, more nuclei, and enlarged F-actin bands were notably identified in the tension stimulation group compared to the no tension stimulation group. To determine the effect of exosomes on osteoclastic activity, we employed a pit formation assay. In response, the area of resorption pits remarkably enlarged in the tension stimulation group (Fig. 2H). Western blotting revealed that expression levels of c-fos, NFATc1, RANK, and NFκB p-P65 induced by RANKL were upregulated in response to exosomes in the tension stimulation group (Fig. 2I). From the results of the histograms coverage rate, number and nuclei of TRAP-positive osteoclasts were significantly increased after the addition of exosomes with tension stimulation. The opposite tendency was observed in the bone resorption area rate (Fig. 2J). RT-qPCR analysis revealed that expression levels of Ctsk, MMP9, and TRAP mRNA induced by RANKL were increased in response to exosomes in the tension stimulation group (Fig. 2K). These results implied that exosomes in the tension stimulation group positively regulate RANKL-induced osteoclastic differentiation and function.

### Circ_0008542 in exosomes upregulates osteoclast differentiation and bone resorption

**Fig. 3:**
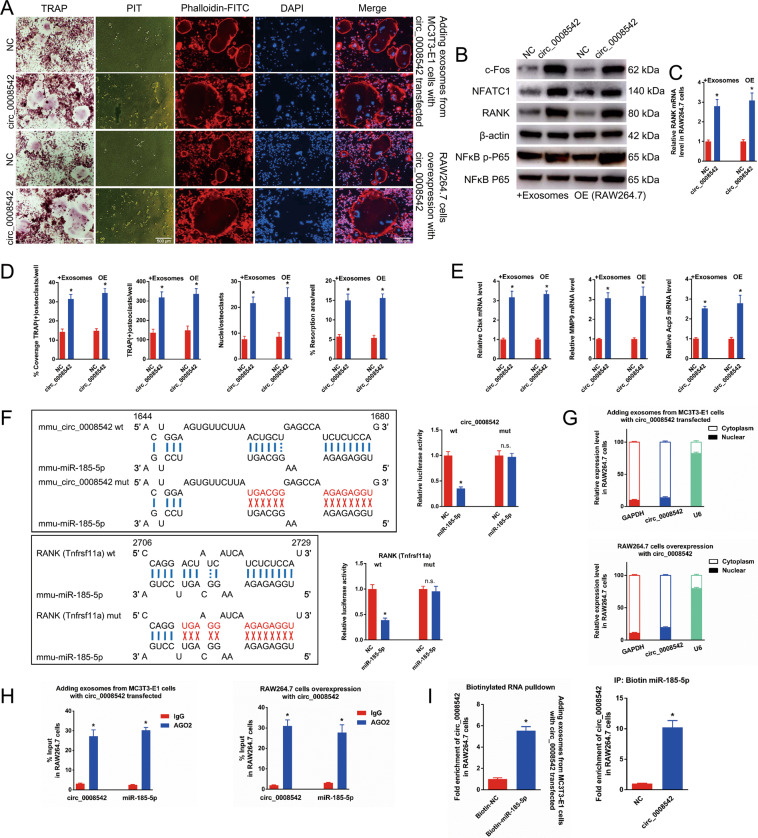
The effects of circ_0008542 on osteoclasts. After transfection of circ_0008542 in RAW264.7 cells or addition of exosomes containing circ_0008542 overexpression in RAW264.7 cells, **A** TRAP staining, pit formation assay, and F-actin band staining were applied to detect osteoclast differentiation and bone resorption ability between the two groups with or without circ_0008542. **B** Protein levels of c-fos, NFATc1, RANK, and NFκB p-P65 in RAW 264.7 cell lysates between two groups with or without circ_0008542 were analyzed by western blot. **C** Relative expression level of RANK in RAW 264.7 cells between two groups with or without circ_0008542. **D** Histograms of coverage rate, number and nuclei of TRAP-positive osteoclasts, and bone resorption area rate between the two groups. **E** Relative expression levels of Ctsk, MMP9, and TRAP in RAW 264.7 cells between the two groups. **F** After construction of the circ_0008542 fragment luciferase reporter or RANK gene fragment luciferase reporter with wild-type or mutant complementary binding sites, relative luciferase activity was detected between the miRNA-185-5p group and the NC group. **G** Cytoplasmic and nuclear fractionation assay was applied to detect localization of circ_0008542. **H** RIP assay was performed to detect the enrichment rate of circ_0008542 and miRNA-185-5p. **I** RNA pulldown assay with 3′-end biotinylated miRNA-185-5p. The binding activities of circ_0008542 to 3′-end biotinylated miRNA-185-5p with circ_0008542 overexpression. Data were representative of three independent experiments expressed as the mean ± SD (*p < 0.05).

We first transfected circ_0008542 in RAW264.7 cells or added exosomes containing circ_0008542 overexpression in RAW264.7 cells. As shown in the results of abovementioned experiments (Fig. 3A–E), circ_0008542 significantly increased osteoclast differentiation and bone resorption compared to the NC groups. Next, the circ_0008542 fragment or RANK gene fragment with wild-type or mutant complementary binding sites was inserted into the luciferase reporter, separately, with miRNA-185-5p mimic and NC constructed. Results showed that the luciferase activity of circ_0008542-wt or RANK-wt was significantly inhibited in the miRNA-185-5p mimic group compared to the NC group (Fig. 3F). In addition, circ_0008542 was predominantly localized in the cytoplasm as evaluated by cytoplasmic and nuclear fractionation assay (Fig. 3G). RNA induced silencing complexes (RISCs) are formed by miRNA ribonucleoprotein complexes (miRNPs), which are present in anti-AGO2 immunoprecipitates. Therefore, anti-AGO2 immunoprecipitates contain miRNAs and their interacting RNA components. RNA immunoprecipitation (RIP) assay revealed that circ_0008542 was enriched in miRNPs containing AGO2 compared to anti-IgG immunoprecipitates (Fig. 3H). RNA pulldown assay proved a significant enrichment of circ_0008542 in the miRNA-185-5p captured fraction compared with the NC group (Fig. 3I).

### Circ_0008542 acts as a miRNA sponge for miR-185-5p and promotes osteoclast-induced bone resorption through the miR-185-5p/RANK axis

We transfected miRNA-185-5p mimic, miRNA-185-5p inhibitor, or si-RANK into RAW264.7 cells. As shown in the results of abovementioned experiments (Fig. S1A–E), either upregulation of miRNA-185-5p or downregulation of RANK inhibited osteoclast differentiation and bone resorption. Combined miRNA-185-5p inhibitor and si-RANK rescued the biological effects caused by miRNA-185-5p inhibitor. Next, we transfected miRNA-185-5p mimic or added exosomes containing circ_0008542 overexpression into RAW264.7 cells. As shown in the results of abovementioned experiments (Fig. S1F–J), combined miRNA-185-5p mimic and circ_0008542 rescued osteoclast differentiation and bone resorption caused by circ_0008542. In addition, we transfected si-RANK then added exosomes (circ_0008542 overexpression from MC3T3-E1 cells) into RAW264.7 cells. The experiment results showed that decreased osteoclast differentiation and bone resorption were notably identified in the circ_0008542+si-RANK group compared to the circ_0008542 group (Fig. S3A–D). These results demonstrated that circ_0008542 acts as a sponge of miRNA-185-5p to promote osteoclast differentiation and bone resorption through the circ_0008542/miRNA-185-5p/RANK axis.

### Only the segment of circ_0008542-9 reveals a high level of m6A methylation

We applied the SRAMP website to predict the abundance of m6A methylation loci of circ_0008542. Ten potential loci were observed in circ_0008542’s overall length (Fig. S2A). To determine the effective m6A methylation segments in circ_0008542, we designed seven pairs of primers that amplified seven sections of the circ_0008542 sequence. From the results of m6A-RT-qPCR, only the circ_0008542-9 segment revealed a high level of m6A methylation (Fig. S2B). To determine the effect of methyltransferase or demethyltransferase on circ_0008542 m6A methylation, we transfected si-METTL3 or ALKBH5 into ME3T3-E1 cells. The previously high levels of m6A methylation of circ_0008542-9 were decreased when disturbed METTL3 or overexpressed ALKBH5 in MC3T3-E1 cells based on m6A-RT-qPCR results (Fig. S2C–F), indicating that both METTL3 and ALKBH5 regulate m6A methylation levels through the segment of circ_0008542-9.

### Inhibiting METTL3 or overexpressing ALKBH5 in MC3T3-E1 cells reverses the effects of osteoclast differentiation and bone resorption induced by adding exosomes with circ_0008542 overexpression

**Fig. 4:**
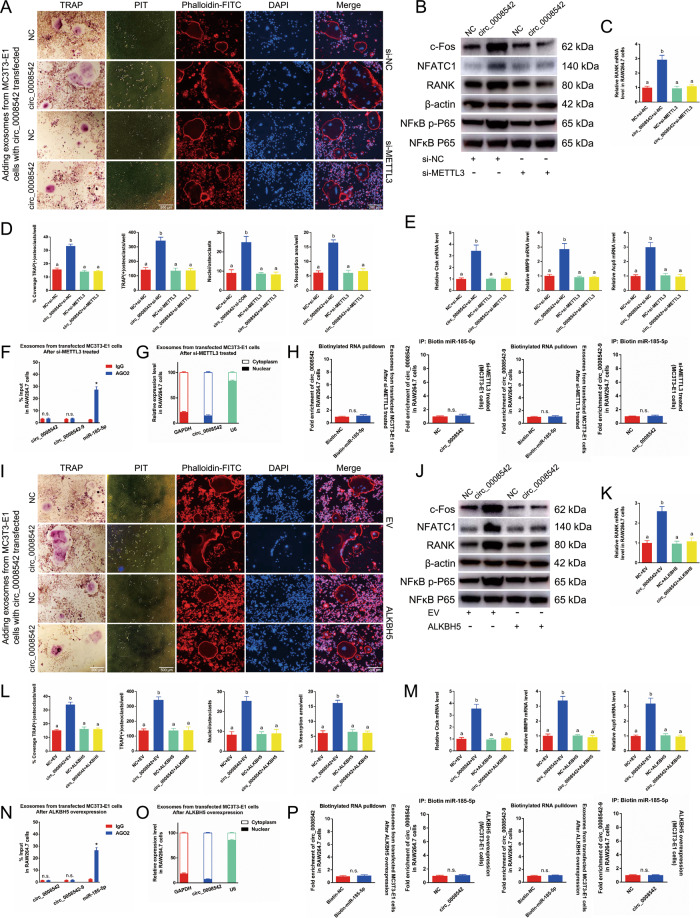
The effects of METTL3 inhibition or ALKBH5 overexpression on the circ_0008542/miR-185-5p/RANK axis. We first transfected circ_0008542 or circ_0008542+si-METTL3 into MC3T3-E1 cells and then added different exosomes containing circ_0008542 overexpression to RAW264.7 cells. **A** TRAP staining, pit formation assay, and F-actin band staining were applied to detect osteoclast differentiation and bone resorption ability among the four groups. **B** Protein levels of c-fos, NFATc1, RANK, and NFκB p-P65 in RAW 264.7 cell lysates among the four groups were analyzed by western blot. **C** Relative expression level of RANK in RAW 264.7 cells among the four groups. **D** Histograms of coverage rate, number and nuclei of TRAP-positive osteoclasts, and bone resorption area rate among the four groups. **E** Relative expression levels of Ctsk, MMP9, and TRAP in RAW 264.7 cells among the four groups. **F** RIP assay was performed to detect the enrichment rate of circ_0008542, circ_0008542-9, and miRNA-185-5p after si-METTL3 treatment. **G** Cytoplasmic and nuclear fractionation assay was applied to detect localization of circ_0008542. **H** RNA pulldown assay with 3′-end biotinylated miRNA-185-5p after si-METTL3 treatment. The binding activities of circ_0008542 or circ_0008542-9 to 3′-end biotinylated miRNA-185-5p with circ_0008542 overexpression. We next transfected circ_0008542 or circ_0008542  +  ALKBH5 into MC3T3-E1 cells and then added different exosomes containing circ_0008542 overexpression to RAW264.7 cells. **I** TRAP staining, pit formation assay, and F-actin band staining were applied to detect osteoclast differentiation and bone resorption ability among the four groups. **J** Protein levels of c-fos, NFATc1, RANK, and NFκB p-P65 in RAW 264.7 cell lysates among the four groups were analyzed by western blot. **K** Relative expression level of RANK in RAW 264.7 cells among the four groups. **L** Histograms of coverage rate, number and nuclei of TRAP-positive osteoclasts, and bone resorption area rate among the four groups. **M** Relative expression levels of Ctsk, MMP9, and TRAP in RAW 264.7 cells among the four groups. **N** RIP assay was performed to detect the enrichment rate of circ_0008542, circ_0008542-9, and miRNA-185-5p after ALKBH5 overexpression. **O** Cytoplasmic and nuclear fractionation assay was applied to detect localization of circ_0008542. **P** RNA pulldown assay with 3′-end biotinylated miRNA-185-5p after ALKBH5 overexpression. The binding activities of circ_0008542 or circ_0008542-9 to 3′-end biotinylated miRNA-185-5p with circ_0008542 overexpression. Data were representative of three independent experiments expressed as the mean ± SD. Different letters (a and b) indicate significant differences among multiple groups (*p* < 0.05).

We transfected circ_0008542 or circ_0008542+si-METTL3/ALKBH5 into MC3T3-E1 cells, and then added different exosomes containing circ_0008542 overexpression to RAW264.7 cells. The same experiments were performed in this part. Results showed that increased osteoclast differentiation and bone resorption were notably identified in the circ_0008542 group but sharply decreased in the circ_0008542+si-METTL3/ ALKBH5 group (Fig. 4A–E, I–M). Meanwhile, the RIP assay demonstrated that circ_0008542 or circ_0008542-9 was not enriched in miRNPs containing AGO2 after si-METTL3/ALKBH5 (Fig. 4F, N). Next, circ_0008542 was predominantly localized in the cytoplasm as evaluated by cytoplasmic and nuclear fractionation assay after si-METTL3/ALKBH5 (Fig. 4G, O). Compared with the NC group the enrichment of circ_0008542 or circ_0008542-9 showed no change in the miRNA-185-5p captured fraction after si-METTL3/ALKBH5 (Fig. 4H, P). In addition, we transfected RANK then added exosomes (circ_0008542 overexpression from MC3T3-E1 cells after si-METTL3/ALKBH5 treated) into RAW264.7 cells. The experiment results showed that increased osteoclast differentiation and bone resorption were notably identified in the circ_0008542+si-METTL3/ALKBH5 + RANK group compared to the circ_0008542+si-METTL3/ALKBH5 group (Fig. S3E–L). It indicated that circ_0008542 m6A methylation level, which is regulated by METTL3 and ALKBH5, is closely related to its function in promoting osteoclast differentiation and bone resorption. Next, after adding exosomes with tension stimulation (Flexcell culture with 20% amplitude/1 Hz/24 h), the same tendency was presented in the condition of miRNA-185-5p mimic transfection or si-METTL3/ALKBH5 (Fig. S4A–I).

### MUT1956 circ_0008542 in exosomes loses its promoting function on osteoclast differentiation and bone resorption

**Fig. 5:**
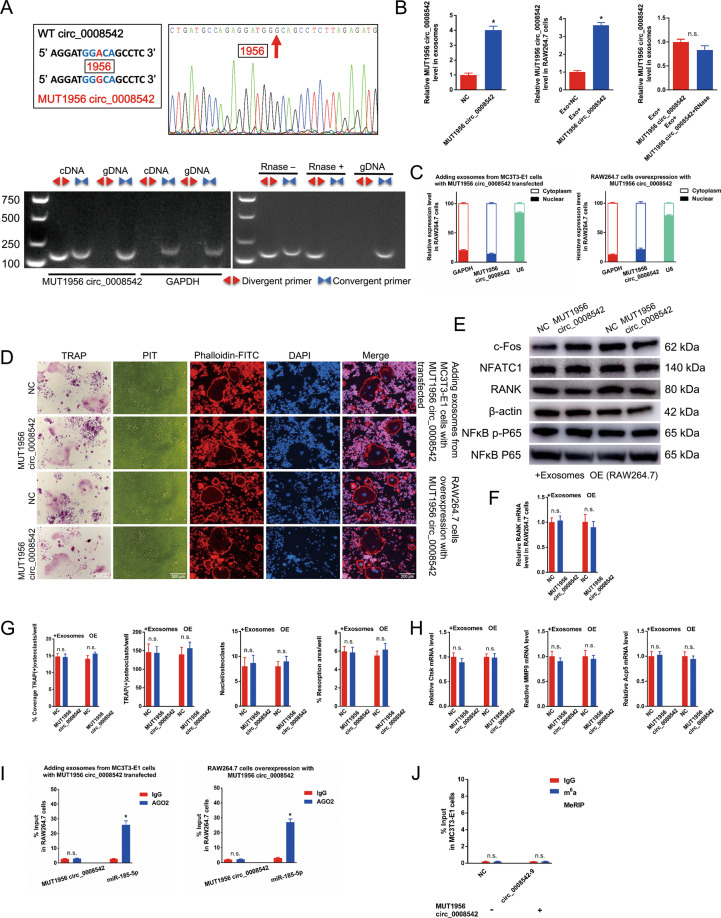
The function of MUT1956 circ_0008542. **A** Schematic diagram shows the mutation site of MUT1956 circ_0008542. Sanger sequencing of MUT1956 circ_0008542. The arrow shows the mutation site. Divergent primers detected MUT1956 circ_0008542 from cDNA by PCR and agarose gel electrophoresis, rather than from gDNA. MUT1956 circ_0008542 from cDNA was amplified with divergent primers and even treated with RNase R digestion, and the opposite results were observed for gDNA. **B** Relative expression level of MUT1956 circ_0008542 in exosomes with or without MUT1956 circ_0008542 overexpression. Relative expression level of MUT1956 circ_0008542 in RAW264.7 cells treated with different exosomes. Relative expression level of MUT1956 circ_0008542 in exosomes with or without RNase R digestion. **C** Cytoplasmic and nuclear fractionation assay was applied to detect localization of MUT1956 circ_0008542. After transfection of MUT1956 circ_0008542 in RAW264.7 cells or addition of exosomes containing MUT1956 circ_0008542 overexpression in RAW264.7 cells. **D** TRAP staining, pit formation assay, and F-actin band staining were applied to detect osteoclast differentiation and bone resorption ability between the two groups with or without MUT1956 circ_0008542. **E** Protein levels of c-fos, NFATc1, RANK, and NFκB p-P65 in RAW 264.7 cell lysates between the two groups with or without MUT1956 circ_0008542 were analyzed by western blot. **F** Relative expression level of RANK in RAW 264.7 cells between the two groups with or without MUT1956 circ_0008542. **G** Histograms of coverage rate, number and nuclei of TRAP-positive osteoclasts, and bone resorption area rate between the two groups. **H** Relative expression levels of Ctsk, MMP9, and TRAP in RAW 264.7 cells between the two groups. **I** RIP assay was performed to detect the enrichment rate of MUT1956 circ_0008542 and miRNA-185-5p. **J** After transfecting MC3T3-E1 cells with MUT1956 circ_0008542, m6A-RT-qPCR assay was performed to detect the enrichment rate of the circ_0008542-9 segment between the anti-m6A group and the anti-IgG group. Data were representative of three independent experiments expressed as the mean ± SD (*p < 0.05).

We next applied site-directed mutagenesis to change the 1956 bp “A” to “G” on circ_0008542. The mutation site matched the sequence from Sanger sequencing (Fig. 5A). In addition, RT-PCR results showed that a single and distinct product of expected size was amplified using divergent primers from only cDNA, while there was no amplification product from gDNA. RNase R digestion results showed that MUT1956 circ_0008542 exhibited a stable feature after RNase R treatment (Fig. 5A, B). After adding exosomes containing MUT1956 circ_0008542 overexpression, expression levels of MUT1956 circ_0008542 increased in exosomes or RAW 264.7 cells as shown by the RT-qPCR results (Fig. 5B). Futhermore, MUT1956 circ_0008542 was predominantly localized in the cytoplasm as evaluated by cytoplasmic and nuclear fractionation assay (Fig. 5C). We next transfected MUT1956 circ_0008542 into RAW264.7 cells or added exosomes containing MUT1956 circ_0008542 overexpression into RAW264.7 cells. The same experiments were performed in this part. Results showed no change of osteoclast differentiation and bone resorption in the MUT1956 circ_0008542 groups (Fig. 5D–H). Meanwhile, RIP assay demonstrated that MUT1956 circ_0008542 was not enriched in miRNPs containing AGO2 compared with anti-IgG immunoprecipitates (Fig. 5I). In addition, the level of m6A methylation of circ_0008542-9 showed no change in the anti-m6A group compared to the anti-IgG group after MUT1956 circ_0008542 treatment based on the m6A-RT-qPCR results (Fig. 5J), indicating that after the mutation of 1956 bp “A” to “G”, circ_0008542 loses its m6A methylation modification which is relevant to its promotion of osteoclast differentiation and bone resorption through the miR-185-5p/RANK axis. Next, in order to exclude the binding efficacy was not altered when the mutation was at 1956 bp, we applied MUT988 circ_0008542 to detect osteoclast function. The mutation at 988 bp was the eighth locus and had a same sequence GGACA with the 1956 bp locus but revealed a low level of m6A methylation. As shown in the results, MUT988 circ_0008542 significantly increased osteoclast differentiation compared to the NC group (Fig. S4J–O).

### ALKBH5 in exosomes displays a clear rescue effect on bone loss induced by circ_0008542 in vivo

**Fig. 6:**
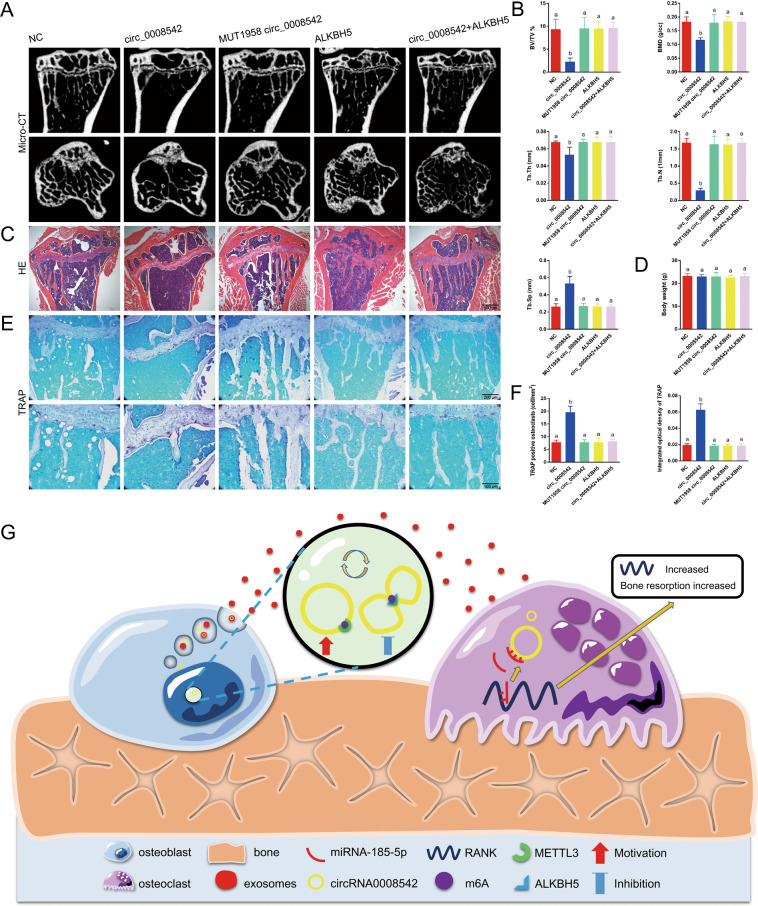
The effects of circ_0008542 in vivo. Circ_0008542, MUT1956 circ_0008542, or ALKBH5 was transfected into MC3T3-E1 cells and collected from cell supernatant exosomes separately or simultaneously according to the different groups. **A**, **C** After 8 weeks of exosome injection, micro-CT, and H&E staining were applied to measure the bone histological parameters. **B**, **D** Histograms of BV/TV, BMD, Tb.Th, Tb.N, Tb.Sp, and body weight among the five groups. **E** TRAP staining was applied to evaluate the osteoclast differentiation and bone resorption ability. **F** Histograms of TRAP positive osteoclast number and integrated optical density of TRAP among the five groups. **G** Schematic diagram of this study. Data were representative of five independent experiments expressed as the mean ± SD. Different letters (a and b) indicate significant differences among multiple groups (*p* < 0.05).

Circ_0008542, MUT1956 circ_0008542, or ALKBH5 was transfected into MC3T3-E1 cells and collected from cell supernatant exosomes separately or simultaneously according to the different groups. Micro-CT and hematoxylin and eosin (H&E) staining were applied to measure bone histological parameters. After 8 weeks of exosome injection, the circ_0008542 group displayed clear bone loss by histology compared to the other four groups, including decreased trabecular bone number, thinner metaphyseal trabecular, and increased trabecular spacing. The circ_0008542 + ALKBH5 group displayed a clear rescue effect on bone loss compared to the circ_0008542 group (Fig. 6A). Compared to the other four groups, the circ_0008542 group observably decreased bone volume/total volume (BV/TV), bone mineral density (BMD), trabecular thickness (Tb.Th), and trabecular number (Tb.N) and significantly increased trabecular separation (Tb.Sp). Compared to the circ_0008542 group, the circ_0008542 + ALKBH5 group observably increased BMD, BV/TV, Tb.Th, and Tb.N and significantly decreased Tb.Sp (Fig. 6B). H&E staining also exhibited the same alteration in mice after exosome injection (Fig. 6C). There was no body weight change in mice among the five groups (Fig. 6D). As expected from TRAP staining, circ_0008542 considerably increased the number of TRAP-positive osteoclasts compared to the other four groups. However, the circ_0008542 + ALKBH5 group observably reversed this phenomenon (Fig. 6E). Several significant differences among the five groups with regard to the number of TRAP positive osteoclasts and integrated optical density of TRAP are presented in the histograms (Fig. 6F). A schematic diagram of this study is presented in Fig. 6G.

## Discussion

Compared to traditional oral implants, immediate implants could shorten the unloaded healing process, improve satisfaction with treatment, and may become the most commonly used treatment. However, clinical studies have shown that this loaded method simultaneously causes marginal bone resorption and increases the failure rate of osseointegration. For example, the previous study proved that after 3 years of follow-up period, the immediate loading group recorded significant vertical bone loss at distal and labial sites than the conventional loading group [33]. The degree of primary stability during immediate loading protocols is dependent on several factors including bone density and quality, implant shape, design and surface characteristics, and surgical technique [34]. Compared with conventional loading, immediate loading is associated with a higher incidence of implants failure [35]. And the risk of early loss of implants in the immediate loading group is higher than that in the delayed loading group [36]. Many clinical researches focus on bone resorption of immediate implants, but few basic researches about the mechanisms between immediate implants and bone resorption especially under tension stimulation. The current dominate view is that micromotion might hinder the proliferation of osteoblasts and lead to the formation of fibrous tissues at the bone-implants interface [34, 37]. But the specific mechanism is still unknown. Therefore, it is important to further study mechanisms of tension stimulus on osteoblasts and osteoclasts at early stage of osseointegration to promote rapid osseointegration around oral implants.

Compared to traditional biomechanical research, this study transformed tension stimulation into circ_0008542, which was proven to be a novel pathogenic molecule in the osseointegration microenvironment. Specifically, exosomes were used as carriers for communication between osteoblasts and osteoclasts. Circ_0008542 is involved in the initiation and progression of disease as an important pathogenic molecule. RNA m6A methylation plays a key role as an important epigenetic modification in the posttranscriptional regulation of circ_0008542 which exerts a molecular sponge effect in osteoclasts through the miRNA-185-5p/RANK axis.

In this study, circ_0008542 was demonstrated to be a novel circular RNA contained in MC3T3-E1 cell exosomes under tension stimulation (Flexcell culture with 20% amplitude/1 Hz/24 h). It is characterized by a head-to-tail splicing form and remains stable under RNase R digestion. In addition, circ_0008542 increased with prolonged tension stimulation time in MC3T3-E1 cells. Therefore, its molecular sponge effect on miR-185-5p was gradually enhanced in RAW264.7 cells. Circ_0008542 gradually upregulates osteoclast differentiation and bone resorption through the miR-185-5p/RANK axis. This phenomenon is a novel molecular mechansim in the osseointegration microenvironment in response to unbalanced stress on the implant neck.

Next, inhibition of METTL3 or overexpression of ALKBH5 in MC3T3-E1 cells reversed the effect of osteoclast differentiation and bone resorption induced by adding exosomes with circ_0008542 overexpression. This means that the regulation of METTL3 or ALKBH5 occurs before circ_0008542 is processed and matured. Once circ_0008542 matured and was contained within exosomes, neither METTL3 nor ALKBH5 influenced its effect on osteoclasts. We postulate that METTL3 or ALKBH5 recognizes the local structure of circ_0008542 through the m6A functional site, changes its spatial structure, and affects the binding efficiency between circ_0008542 and miRNA-185-5p, thus impacting RANK expression and osteoclast function. If circ_0008542 is not processed by METTL3 or ALKBH5 during maturation, its spatial structure is not conducive to binding with miRNA-185-5p, thus losing its sponge effect. Pan T and Parisien M reached a similar conclusion in a previous study. M6A alters the local structure in mRNA and lncRNA to facilitate binding of heterogeneous nuclear ribonucleoprotein C (hnRNP C). Specifically, the 2577 m6A residue destabilizes lncRNA MALAT1 hairpin-stem to make its opposing U-tract more single-stranded or accessible, enhancing its interaction with hnRNP C. They term this mechanism that regulates RNA–protein interactions through m6A-dependent RNA structural remodeling an “m6A-switch” [38]. In addition, Tavazoie SF et al. demonstrated that the m6A mark acts as a key posttranscriptional modification that promotes the processing of primary microRNAs. Specifically, pri-miRNAs are marked by METTL3-dependent m6A modification, and METTL3 expression is required for the appropriate processing of most pri-miRNAs to mature miRNAs. Therefore, the m6A mark plays an important role in the nucleus, allowing the microprocessor complex to recognize its specific secondary structures with methylation sites at the same time, as opposed to unintended secondary structures in mRNA [39]. The MUT1956 circ_0008542 study validated this conclusion. After the mutation of 1956 bp “A” to “G” on circ_0008542, MUT1956 circ_0008542 in exosomes lost its promoting function on osteoclast differentiation and bone resorption. Thus, even without interference of METTL3 or ALKBH5 during the process of circRNA maturation, mutation of the m6A functional site directly results in loss of its biological effects. Therefore, based on the above conclusions, m6A methylation determines the molecular sponge effect of circ_0008542. METTL3 or ALKBH5 may alter the spatial structure of genes (circ_0008542) through specific m6A functional sites, facilitating their binding with target genes without changing their nucleotide base sequence. The m6A functional site is the core of relevant biological effects. This “m6A-switch” at circ_0008542 1956 bp is closely related to osteoclast differentiation and bone resorption in osteoclast precursor cells. METTL3 or ALKBH5 appears to be a node that controls the “m6A-switch” to varying degrees.

From the results of the in vivo study, ALKBH5 overexpression in exosomes clearly rescued the bone loss induced by circ_0008542 after 8 weeks of exosome injection. Specifically, ALKBH5 acts on the circ_0008542 1956 bp “m6A-switch” through demethylation, and makes circ_0008542 unsuitable for binding to the miR-185-5p/RANK axis. After decreasing the molecular sponge effect of circ_0008542, osteoclast differentiation and bone resorption were also reduced. Combined with the clinical problem that needs to be solved in this study, we think that oral implants are widely used, and failure of osseointegration is the core reason for failure of oral implants. Therefore, the potential value of this study provides methods to enhance the resistance of immediate implants through use of exosomes releasing ALKBH5.

## Materials and Methods

### Cells culture, antibodies, Flexcell tension system application and ethics statement

Murine RAW264.7 monocytic and MC3T3-E1 cell lines were purchased from the Shanghai Cell Center (Shanghai, China). Recombinant soluble mouse RANKL was purchased from R&D Systems (Minneapolis, USA). Specific antibodies against NFκB p65, phospho-NFκB p65, horseradish peroxidase-conjugated goat anti-rabbit, NFATc1, and RANK were obtained from Cell Signaling Technology (Shanghai, China). Specific antibodies against HSP70, TSG101, CD63, TFIIB, Lamin A/C, c-fos, METTL3, ALKBH5, β-actin, and GAPDH were obtained from Abcam (Shanghai, China). All animal experiments in this study were conducted according to the Guidelines for Animal Experimentation of Shanghai Jiao Tong University (Ethics number: SH9H-2020-A41-1). We isolated BMSCs from mice for cultured. Briefly, we first isolated the tibiae and femur from 8-week-old female C57BL/6 mice (Animal Center of Shanghai Jiao Tong University, Shanghai, China). D-Hanks was used to completely wash marrow cells. The lavage was first passed through a cell strainer and was then centrifuged (300 g for 5 min). A single cell suspension was collected in αMEM supplemented with 10% FBS and 1% penicillin/streptomycin. MC3T3-E1 cells were seeded into 6-well BioFlex Culture Plate-Untreated (BioFLEX, USA). The medium were replaced with fresh basal medium when the cell density reached 80–90% confluency. Then, cells were subjected to cyclic mechanical tension (20% amplitude/1 Hz/6–48 h) using a FX-5000T Flexcell Tension system (Flexcell, USA). Cells and supernatants were subsequently collected for further analysis.

### Exosome isolation, transmission electron microscopy, and particle size analysis

Cell supernatants were centrifuged at 2000x*g* for 15 min at 4 °C to exclude cell debris. Supernatants were filtered through 0.22 μm filters (Millipore, USA) and then centrifuged at 110,000x*g* for 30 min in Amicon Ultra -3 KDa (Millipore, USA). Pellets were resuspended with appropriate PBS and stored at −80 °C. Freshly prepared exosomes were resuspended in 100 μl of 2% PFA, absorbed on Formvar-carbon coated EM grids, washed with PBS, fixed with 1% glutaraldehyde, and washed with water. Thereafter, grid were stained with 4% uranyl-oxalate solution, embedded in 1% methyl cellulose-UA, and observed under electron microscopy at 80 kV. A ZetaView PMX 110 instrument (Particle Metrix, Germany) was used to analyze the distribution of exosome size. Freshly prepared exosome samples were resuspended in PBS and measured according to the manufacturer’s instructions.

### ALP staining and ARS staining

ALP staining was performed using a BCIP/NBT staining kit (Beyotime, China). After osteogenic induction for 7 days, cells were fixed and ALP staining was performed following the manufacturer’s instructions. Mineralized nodule formation was determined by ARS staining. After osteogenic incubation for 21 days, cells were fixed and stained with 0.1% ARS (Sigma-Aldrich, USA) for 20 min.

### Osteoclast differentiation, TRAP staining, F-actin band staining, and pit formation assay

RAW264.7 cells were seeded into 48-well plates and cultured in DMEM containing 10% FBS and 1% penicillin/streptomycin. Cells were subjected to treatments according to experimental requirements. Cells were then stimulated with RANKL (20 ng/ml) for 7 days. Culture medium was replaced with fresh medium every other day. After 7 days, TRAP staining was used to evaluate osteoclast differentiation. Cells were fixed and subjected to TRAP staining. Briefly, cells were submerged in a mixture of 3.0 mg naphthol AS-BI phosphate, 18 mg red violet LB salt, and 100 mML (+) tartaric acid (0.36 g) diluted in 30 ml of 0.1 M sodium acetate buffer (pH 5.0) for 15 min at 37 °C. Multinucleated TRAP-positive cells with at least three nuclei were scored as osteoclasts. RAW264.7 cells were seeded into 48-well plates and cultured for 7 days as previously described. Cells were fixed and stained for F-actin band staining. Briefly, cells were submerged in 5 μl/ml phalloidin (Yeasen, China) for 30 min after treatment with 0.5% Triton X-100 for 5 min. The pit formation assay was performed using Corning osteo assay surface multiple well plates (Corning, USA). RAW264.7 cells were seeded into 96-well plates and cultured for 10 days as previously described. Next, plates were stained with Von Kossa to increase the contrast between pits and surface coating and observed under a light microscope.

### Western blot analysis

RIPA lysis buffer was used to extract total protein from cultured cells. Equal quantities of proteins were separated using SurePAGE gel (Genscript, USA) electrophoresis and transferred onto polyvinylidene difluoride (PVDF) membranes (Millipore, USA). PVDF membranes were incubated with primary antibodies (TFIIB, Lamin A/C, HSP70, TSG101, CD63, c-fos, NFATc1, RANK, NFκB P65, and phospho-NF-κB P65) overnight at 4 °C. After washing, PVDF membranes were incubated with secondary antibodies. Protein bands were visualized using ECL chemiluminescence reagent (Millipore, USA).

### RT-qPCR analysis

Total RNA was extracted using TRIzol reagent (Takara, Japan) and was reverse transcribed using the PrimeScript^TM^ RT regent Kit with gDNA Eraser and Mir-X miRNA First-Strand Synthesis Kit (Takara, Japan). Primer sequences were designed and synthetized by Sangon Biotech (Table S3). RT-qPCR was conducted with SYBR Premix Ex Taq^TM^ II (Takara, Japan). Relative expression levels of the target genes were calculated by the 2^-∆∆Ct^ method. GAPDH or U6 was used for normalization, and the data were compared to normalized control values.

### RNA isolation of nuclear and cytoplasmic fractions

RNA isolation of nuclear and cytoplasmic fractions was performed using the Nuclear/Cytoplasmic Isolation Kit (Biovision, USA). Expression levels of circ_0008542, U6, and GAPDH were analyzed by RT-qPCR.

### RNA oligoribonucleotides

The RNA oligoribonucleotides used in this study, including miR-185-5p mimic, miR-185-5p inhibitor, small interfering RNAs (siRNAs) targeting RANK (si-RANK), and METTL3 (si-METTL3), were purchased from Ribo Life Science Co. (Shanghai, China). Lentiviral constructs containing circ_0008542, MUT1956 circ_0008542, and MUT988 circ_0008542 were purchased from Geneseed Biotech Co. (Guangzhou, China). The lentiviral construction of ALKBH5 and RANK were purchased from Vigene Biosciences Co. (Jinan, China). Sequences of these RNA oligoribonucleotides are listed in Table S4.

### Luciferase reporter assay

Luciferase reporters were generated by cloning circ_0008542, mutant-circ_0008542, wild-type-RANK-3′UTR, or mutant-RANK-3′UTR into GP-miRGLO vectors. Briefly, luciferase reporter plasmids were transfected into RAW264.7 cells together with miR-NC or miR-185-5p mimic using Lipofectamine 3000. After transfection for 24 h, Renilla and firefly luciferase activities were measured separately using the Dual Luciferase Reporter Assay System (Promega, USA) following the manufacturer’s instructions. Renilla luciferase was used to normalize firefly luciferase activity to evaluate reporter translation efficiency.

### RNA immunoprecipitation (RIP)

RIP assay was performed with a Magna RIP Kit (Millipore, USA) according to the manufacturer’s protocol. Briefly, magnetic beads were mixed with anti-Argonaute 2 (AGO2) (Abcam, China) or anti-IgG (Cell Signaling Technology, China) before the addition of cell lysates. After the protein beads were removed, RNAs of interest were eluted from the immunoprecipitated complex and purified for further analysis using RT-qPCR. Relative enrichment was normalized to the input.

### M6A immunoprecipitation (MeRIP)

MeRIP assay was conducted with the Magna MeRIP™ m6A Kit (Millipore, USA) to determine m6A modification of individual transcripts. In brief, total RNA was isolated from pretreated cells and randomly fragmented into a size of 100 nucleotides. RNA samples were then immunoprecipitated with magnetic beads precoated with anti-m6A antibody (Millipore, USA) or anti-mouse IgG (Millipore). N6-methyladenosine 5′-monophosphate sodium salt was applied to elute the m6A-modified RNA fragments for further RT-qPCR analysis. Specific primers were designed for RT-qPCR analysis according to the SRAMP website (m6A loci predictor http://www.cuilab.cn/sramp/) and were listed in Table S5. The relative enrichment of m6A was normalized to the input.

### RNA pulldown assay

The 3′-end biotinylated miRNA-185-5p mimics (Ribio, China) was constructed for RNA pulldown. Streptavidin-coated magnetic beads (Invitrogen, USA) were used to incubate the probe at 25 °C for 1 h to generate probe-coated magnetic beads. The cells were harvested in a lysis buffer and the lysate was incubated with probe-coated magnetic beads at 37 °C for 4 h with constant rotation. After incubation, three washes with lysis buffer were performed and RNA was extracted using TRIzol reagent. The abundance of circ_0008542 in bound fraction was evaluated by RT-qPCR analysis.

### Animals and exosome injection

Eight-week-old female C57BL/6 mice were used in this study. All mice were randomly distributed into five groups: NC, where rats were injected with exosomes containing mock vehicle into the tail vein for 8 weeks (exosomes 100 μg/week, for 8 weeks, *n* = 5); circ_0008542, where rats were injected with exosomes containing circ_0008542 into the tail vein for 8 weeks (same dose, *n* = 5); MUT1956 circ_0008542, where rats were injected with exosomes containing MUT1956 circ_0008542 into the tail vein for 8 weeks (same dose, *n* = 5); ALKBH5, where rats were injected with exosomes containing ALKBH5 into the tail vein for 8 weeks (same dose, *n* = 5), and circ_0008542 + ALKBH5, where rats were injected with exosomes containing circ_0008542 and ALKBH5 into the tail vein for 8 weeks (same dose, *n* = 5). Circ_0008542, MUT1956 circ_0008542, or ALKBH5 was transfected into MC3T3-E1 cells according to the corresponding experimental groups.

### Micro CT, histological detection, and TRAP staining

Tibiae were scanned using Skyscan 1176 Micro-CT (Bruker, USA) with a scanning resolution of 9 μm, a voltage of 50 kV, and a current of 450 μA. Trabecular bone data were obtained at a region of interest along the long axis of the proximal tibiae and 1–3 mm away from the growth plate. The bone histomorphometry parameter analysis included BV/TV, BMD, Tb.Th, Tb.N, and Tb.Sp. Serial sections were made for subsequent histological analysis. H&E staining was applied to assess histological alterations. TRAP staining was performed as previously described.

### Statistical analysis

Data analyses were performed using SAS version 9.4 (SAS Institute, USA). Normal tests were performed to know the normality of continuous data before appropriate ways of statistical description or analysis were chosen. Parametric test of Student’s *t*-test or one-way ANOVA were used if the data were normally distributed. Instead, nonparametric test of Wilcoxon rank sum test or Kruskal–Wallis (*H*-test) would be used if the data did not meet the requirements of parametric test. *P* < 0.05 was considered statistically significant.

## Supplementary information

Supplementary tables and figures

S1

S2

S3

S4
