# Supplementary material for: Circ_0008542 in osteoblast exosomes promotes osteoclast-induced bone resorption through m6A methylation
Source: Cell Death Dis. 2021 Jun 18;12(7):628. doi: 10.1038/s41419-021-03915-1 (PMC8213782; doi:10.1038/s41419-021-03915-1)
Supplement: Supplementary file 1 — Supplementary tables and figures [file 41419_2021_3915_MOESM1_ESM.docx]

**Supplementary tables and figures**

**Figure S1.** **The molecular sponge effects of circ_0008542.** After transfection with the miRNA-185-5p mimic, miRNA-185-5p inhibitor or si-RANK in RAW264.7 cells, **(A)** TRAP staining, pit formation assay and F-actin band staining were applied to detect osteoclast differentiation and bone resorption ability among the five groups. **(B)** Protein levels of c-fos, NFATc1, RANK and NFκB p-P65 in RAW 264.7 cell lysates among the five groups were analyzed by western blot. **(C)** Relative expression level of RANK in RAW 264.7 cells among the five groups. **(D)** Histograms of coverage rate, number and nuclei of TRAP-positive osteoclasts and bone resorption area rate among the five groups. **(E)** Relative expression levels of Ctsk, MMP9 and TRAP in RAW 264.7 cells among the five groups. After transfection with the miRNA-185-5p mimic or the addition of exosomes containing circ_0008542 overexpression to RAW264.7 cells, **(F)** TRAP staining, pit formation assay and F-actin band staining were applied to detect osteoclast differentiation and bone resorption ability among the four groups. **(G)** Protein levels of c-fos, NFATc1, RANK and NFκB p-P65 in RAW 264.7 cell lysates among the four groups were analyzed by western blot. **(H)** Relative expression level of RANK in RAW 264.7 cells among the four groups. **(I)** Histograms of coverage rate, number and nuclei of TRAP-positive osteoclasts and bone resorption area rate among the four groups. **(J)** Relative expression levels of Ctsk, MMP9 and TRAP in RAW 264.7 cells among the four groups. Data are representative of three independent experiments expressed as the mean ± SD. Different letters (a, b and c) indicate significant differences among multiple groups (p < 0.05).

**Figure S2. M6A methylation level on circ_0008542. (A)** The potential m6A methylation loci of circ_0008542 on the SRAMP website. **(B)** M6A-RT-qPCR assay was performed to detect the enrichment rate of 7 segments of circ_0008542 between the anti-m6A group and the anti-IgG group. **(C, D)** After transfection with si-Mettl3 or ALKBH5 in ME3T3-E1 cells, the mRNA and protein expression levels of about Mettl3 or ALKBH5 were detected by RT-qPCR and western blot, respectively. **(E, F)** After transfected si-Mettl3 or ALKBH5 in ME3T3-E1 cells, m6A-RT-qPCR assay was performed to detect the enrichment rate of the circ_0008542-9 segment between the anti-m6A group and the anti-IgG group. Data are representative of three independent experiments expressed as the mean ± SD (*p < 0.05).

**Figure S3.** **The rescue assays caused by circ_0008542 and si-METTL3/ALKBH5 transfection.**

After adding exosomes with circ_0008542 overexpression and combined with si-RANK transfected in RAW264.7 cells, **(A)** TRAP staining, pit formation assay and F-actin band staining were applied to detect osteoclast differentiation and bone resorption ability between the two groups. **(B)** Protein levels of c-fos, NFATc1, RANK and NFκB p-P65 in RAW 264.7 cell lysates between the two groups were analyzed by western blot. **(C)** Histograms of coverage rate, number and nuclei of TRAP-positive osteoclasts and bone resorption area rate between the two groups. **(D)** Relative expression levels of Ctsk, MMP9, TRAP and RANK in RAW 264.7 cells between the two groups. After RANK overexpression combined with circ_0008542 and si-METTL3/ALKBH5 transfected in RAW264.7 cells, **(E, I)** TRAP staining, pit formation assay and F-actin band staining were applied to detect osteoclast differentiation and bone resorption ability between the two groups. **(F, J)** Protein levels of c-fos, NFATc1, RANK and NFκB p-P65 in RAW 264.7 cell lysates between the two groups were analyzed by western blot. **(G, K)** Histograms of coverage rate, number and nuclei of TRAP-positive osteoclasts and bone resorption area rate between the two groups. **(H, L)** Relative expression levels of Ctsk, MMP9, TRAP and RANK in RAW 264.7 cells between the two groups. Data are representative of three independent experiments expressed as the mean ± SD (*p < 0.05).

**Figure S4.** **The same tendency presented by adding exosomes from tension stimulation ME3T3-E1 cells.**

After adding exosomes with tension stimulation (Flexcell culture with 20% amplitude/1 Hz/24 h) and combined with the miRNA-185-5p mimic transfected in RAW264.7 cells, **(A, B)** TRAP staining was applied to detect osteoclast differentiation among the four groups. **(C)** Relative expression levels of Ctsk, MMP9, TRAP and RANK in RAW 264.7 cells among the four groups. As shown in the results, tension stimulation combined with miRNA-185-5p mimic rescued osteoclast differentiation caused by only tension stimulation. We next transfected si-METTL3/ALKBH5 into MC3T3-E1 cells and treated with tension stimulation (Flexcell culture with 20% amplitude/1 Hz/24 h). Then we added different exosomes to RAW264.7 cells. **(D, E, G, H)** TRAP staining was applied to detect osteoclast differentiation among the four groups. **(F, I)** Relative expression levels of Ctsk, MMP9, TRAP and RANK in RAW 264.7 cells among the four groups. As shown in the results, tension stimulation combined with si-METTL3/ALKBH5 rescued osteoclast differentiation caused by only tension stimulation. The function of MUT988 circ_0008542. **(J)** Sanger sequencing of MUT988 circ_0008542. The arrow shows the mutation site. **(K)** Relative expression level of MUT988 circ_0008542 in exosomes or in RAW264.7 cells. Relative expression level of MUT988 circ_0008542 in exosomes with or without RNase R digestion. **(L)** Divergent primers detected MUT988 circ_0008542 from cDNA by PCR, rather than from gDNA. MUT988 circ_0008542 from cDNA was amplified with divergent primers and even treated with RNase R digestion, and the opposite results were observed for gDNA. **(M, N)** TRAP staining was applied to detect osteoclast differentiation between the two groups. **(O)** Relative expression levels of Ctsk, MMP9, TRAP and RANK in RAW 264.7 cells between the two groups. As shown in the results, MUT988 circ_0008542 significantly increased osteoclast differentiation compared to the NC group. Data are representative of three independent experiments expressed as the mean ± SD (*p < 0.05). Different letters (a, b, c, d and e) indicate significant differences among multiple groups (p < 0.05).

**Table S1. Differentially expressed circRNAs.**

| **circRNA_ID** |  | **Locus** |  | **With tension stimulation_CPM** | **Without tension**  **stimulation_CPM** |
| --- | --- | --- | --- | --- | --- |
| mmu_circ_0000081 |  | chr1:139039999-139062971:+ | | 14.14168287 | 3.921492161 |
| chr1:163933478-163934817:+ | | chr1:163933478-163934817:+ | | 13.65673975 | 3.921492161 |
| mmu_circ_0008505 |  | chr1:179999361-180040200:+ | | 14.2548022 | 3.921492161 |
| mmu_circ_0000125 |  | chr1:181923559-181961919:- |  | 7.639764159 | 3.921492161 |
| mmu_circ_0008542 |  | chr1:186736115-186739863:- |  | 14.5490431 | 3.921492161 |
| mmu_circ_0000132 |  | chr1:190871591-190899571:- |  | 3.921492161 | 12.7289389 |
| chr1:32333465-32472765:+ |  | chr1:32333465-32472765:+ |  | 13.02024989 | 3.921492161 |
| chr1:43797244-43807377:- |  | chr1:43797244-43807377:- |  | 11.85361031 | 3.921492161 |
| chr1:71618578-71626246:- |  | chr1:71618578-71626246:- |  | 13.97006944 | 3.921492161 |
| chr1:74681299-74696812:+ |  | chr1:74681299-74696812:+ |  | 3.921492161 | 12.67017937 |
| chr1:78121577-78132433:- |  | chr1:78121577-78132433:- |  | 3.921492161 | 12.7668231 |
| mmu_circ_0000057 |  | chr1:87364103-87380008:+ |  | 16.09213007 | 13.42961648 |
| mmu_circ_0001932 |  | chr10:108268571-108274188:+ | | 13.33523476 | 3.921492161 |
| mmu_circ_0001983 |  | chr10:117099942-117102198:- | | 14.42157734 | 3.921492161 |
| mmu_circ_0002037 |  | chr10:12621190-12688427:- |  | 3.921492161 | 14.02958574 |
| mmu_circ_0000153 |  | chr10:25220610-25289730:- |  | 3.921492161 | 6.720658265 |
| mmu_circ_0002168 |  | chr10:34001136-34012137:- |  | 3.921492161 | 9.526839552 |
| mmu_circ_0002262 |  | chr10:50748805-50754122:+ |  | 14.08161335 | 3.921492161 |
| mmu_circ_0002433 |  | chr10:77630725-77630894:+ |  | 3.921492161 | 9.978166325 |
| mmu_circ_0000193 |  | chr10:82368570-82383844:+ |  | 15.06555951 | 3.921492161 |
| chr10:88124807-88140096:- |  | chr10:88124807-88140096:- |  | 3.921492161 | 13.06929774 |
| mmu_circ_0000330 |  | chr11:104368679-104378826:- | | 3.921492161 | 12.38363624 |
| chr11:107632171-107637927:+ | | chr11:107632171-107637927:+ | | 3.921492161 | 12.82184703 |
| mmu_circ_0002788 |  | chr11:117290397-117291036:+ | | 14.50398416 | 3.921492161 |
| mmu_circ_0000245 |  | chr11:22053431-22068506:- |  | 3.921492161 | 14.02177334 |
| chr11:23289757-23291631:+ | | chr11:23289757-23291631:+ |  | 7.639764159 | 3.921492161 |
| mmu_circ_0002977 |  | chr11:31055457-31061586:+ |  | 15.08098277 | 3.921492161 |
| mmu_circ_0000254 |  | chr11:43704533-43705477:+ |  | 3.921492161 | 13.7176886 |
| chr11:60460786-60462634:+ | | chr11:60460786-60462634:+ |  | 3.921492161 | 13.73690332 |
| chr11:8538002-8579121:- |  | chr11:8538002-8579121:- |  | 3.921492161 | 12.14558555 |
| mmu_circ_0003507 |  | chr11:86661122-86665878:- |  | 9.871223444 | 12.8573973 |
| mmu_circ_0003646 |  | chr12:100595814-100619641:- | | 14.94430913 | 3.921492161 |
| chr12:112963509-112979757:- | | chr12:112963509-112979757:- | | 15.14107802 | 3.921492161 |
| mmu_circ_0000425 |  | chr12:116317829-116324210:+ | | 3.921492161 | 12.38363624 |
| chr12:35078899-35098313:+ | | chr12:35078899-35098313:+ |  | 3.921492161 | 7.613111222 |
| chr12:65136749-65140890:- |  | chr12:65136749-65140890:- |  | 3.921492161 | 13.39374685 |
| chr12:70990484-70993080:+ | | chr12:70990484-70993080:+ |  | 14.33417676 | 3.921492161 |
| mmu_circ_0004195 |  | chr12:75485131-75515798:- |  | 15.08098277 | 3.921492161 |
| mmu_circ_0004257 |  | chr12:83574631-83575136:- |  | 9.55511893 | 3.921492161 |
| chr13:24064576-24099663:- |  | chr13:24064576-24099663:- |  | 3.921492161 | 8.160366165 |
| chr13:45795508-45956575:- |  | chr13:45795508-45956575:- |  | 12.33734843 | 3.921492161 |
| mmu_circ_0000482 |  | chr13:84251229-84260240:+ |  | 3.921492161 | 10.21583614 |
| chr14:21774962-21780181:+ | | chr14:21774962-21780181:+ |  | 14.48091572 | 3.921492161 |
| mmu_circ_0000510 |  | chr14:24190317-24192954:- |  | 8.583889358 | 3.921492161 |
| mmu_circ_0000585 |  | chr15:35072429-35115645:- |  | 3.921492161 | 12.90913226 |
| mmu_circ_0005625 |  | chr15:38672856-38683009:+ |  | 3.921492161 | 12.8573973 |
| mmu_circ_0005665 |  | chr15:41797473-41826052:+ |  | 3.921492161 | 12.74800535 |
| mmu_circ_0000599 |  | chr15:50846013-50846942:- |  | 10.70880892 | 3.921492161 |
| mmu_circ_0000604 |  | chr15:62107147-62107465:+ |  | 3.921492161 | 10.68020631 |
| chr16:13685061-13699075:+ | | chr16:13685061-13699075:+ |  | 9.871223444 | 3.921492161 |
| mmu_circ_0000663 |  | chr16:13697332-13699075:+ |  | 3.921492161 | 13.88204946 |
| mmu_circ_0006095 |  | chr16:17015897-17018475:+ |  | 11.12057737 | 3.921492161 |
| chr16:17764383-17771869:+ | | chr16:17764383-17771869:+ |  | 14.5490431 | 3.921492161 |
| mmu_circ_0000673 |  | chr16:18894773-18899457:+ |  | 13.91949201 | 3.921492161 |
| mmu_circ_0006156 |  | chr16:21434686-21435821:+ |  | 3.921492161 | 13.1857451 |
| chr16:22248435-22259261:- |  | chr16:22248435-22259261:- |  | 8.583889358 | 3.921492161 |
| chr16:35438112-35466657:- |  | chr16:35438112-35466657:- |  | 3.921492161 | 12.8037379 |
| mmu_circ_0006462 |  | chr16:91782160-91793767:+ |  | 13.43462941 | 3.921492161 |
| chr16:91793606-91812153:+ | | chr16:91793606-91812153:+ |  | 3.921492161 | 13.15750251 |
| mmu_circ_0000713 |  | chr16:94383911-94393174:+ |  | 7.639764159 | 3.921492161 |
| mmu_circ_0006510 |  | chr16:96151812-96154195:+ |  | 3.921492161 | 12.33402673 |
| mmu_circ_0006523 |  | chr16:97835577-97839968:- |  | 14.26833828 | 3.921492161 |
| mmu_circ_0000735 |  | chr17:26739542-26743131:+ |  | 3.921492161 | 12.28265039 |
| mmu_circ_0006764 |  | chr17:42845325-42852528:- |  | 3.921492161 | 8.160366165 |
| chr17:46262401-46282439:- |  | chr17:46262401-46282439:- |  | 3.921492161 | 13.06929774 |
| chr17:67849052-67864625:- |  | chr17:67849052-67864625:- |  | 9.149735463 | 3.921492161 |
| mmu_circ_0006964 |  | chr17:71426154-71456500:- |  | 14.14168287 | 3.921492161 |
| mmu_circ_0007004 |  | chr17:74635762-74640342:+ |  | 14.09686683 | 3.921492161 |
| mmu_circ_0000819 |  | chr17:80044493-80053641:- |  | 14.0348607 | 13.17169292 |
| chr17:80278222-80281428:- |  | chr17:80278222-80281428:- |  | 15.14841692 | 3.921492161 |
| chr17:84993679-85005566:+ | | chr17:84993679-85005566:+ |  | 14.12689834 | 3.921492161 |
| chr18:12841183-12882277:- |  | chr18:12841183-12882277:- |  | 14.24113791 | 3.921492161 |
| chr18:34261004-34306379:+ | | chr18:34261004-34306379:+ |  | 3.921492161 | 12.54495336 |
| mmu_circ_0000860 |  | chr18:35152600-35154540:+ |  | 3.921492161 | 10.32143776 |
| chr18:35317841-35418195:- |  | chr18:35317841-35418195:- |  | 3.921492161 | 6.720658265 |
| mmu_circ_0007495 |  | chr18:73657194-73678039:- |  | 14.22734297 | 3.921492161 |
| mmu_circ_0007530 |  | chr18:77097248-77105889:+ |  | 14.11196073 | 3.921492161 |
| mmu_circ_0000903 |  | chr18:79086550-79087180:- |  | 12.98788822 | 3.921492161 |
| mmu_circ_0007562 |  | chr18:80891814-80917878:- |  | 9.55511893 | 3.921492161 |
| chr19:26696175-26720947:+ | | chr19:26696175-26720947:+ |  | 9.149735463 | 3.921492161 |
| chr19:3602234-3605494:- |  | chr19:3602234-3605494:- |  | 13.52761541 | 3.921492161 |
| mmu_circ_0007762 |  | chr19:38865615-38882774:+ |  | 3.921492161 | 12.45499154 |
| chr19:44941458-44943519:+ | | chr19:44941458-44943519:+ |  | 9.149735463 | 3.921492161 |
| chr19:44966966-44969134:+ | | chr19:44966966-44969134:+ |  | 3.921492161 | 11.71287691 |
| chr19:45456532-45471104:+ | | chr19:45456532-45471104:+ |  | 3.921492161 | 11.75117967 |
| mmu_circ_0000955 |  | chr19:45630864-45640521:- |  | 14.30820079 | 3.921492161 |
| mmu_circ_0000962 |  | chr19:56570415-56576284:+ |  | 14.8752188 | 3.921492161 |
| mmu_circ_0000964 |  | chr19:57378548-57379175:+ |  | 3.921492161 | 13.26729975 |
| mmu_circ_0000967 |  | chr19:57751629-57777945:+ |  | 13.83104463 | 3.921492161 |
| mmu_circ_0000968 |  | chr19:57751629-57797503:+ |  | 14.35969328 | 3.921492161 |
| mmu_circ_0001052 |  | chr2:104470748-104471847:- |  | 14.74572751 | 3.921492161 |
| mmu_circ_0009211 |  | chr2:118630944-118631899:+ | | 7.639764159 | 3.921492161 |
| chr2:120392180-120397447:- | | chr2:120392180-120397447:- |  | 14.66615549 | 3.921492161 |
| chr2:127019285-127037859:+ | | chr2:127019285-127037859:+ | | 3.921492161 | 12.82184703 |
| mmu_circ_0001076 |  | chr2:140008698-140057499:- |  | 13.08287367 | 3.921492161 |
| chr2:142756088-142758909:- | | chr2:142756088-142758909:- |  | 3.921492161 | 13.98206387 |
| mmu_circ_0009648 |  | chr2:179921151-179939954:- |  | 12.88620549 | 3.921492161 |
| mmu_circ_0000989 |  | chr2:23537327-23545579:- |  | 14.37228428 | 3.921492161 |
| mmu_circ_0009774 |  | chr2:27452832-27454504:- |  | 3.921492161 | 9.121646537 |
| chr2:29471292-29500326:+ |  | chr2:29471292-29500326:+ |  | 12.48378096 | 3.921492161 |
| chr2:30426398-30439764:+ |  | chr2:30426398-30439764:+ |  | 15.26778427 | 3.921492161 |
| mmu_circ_0009830 |  | chr2:31183579-31188813:+ |  | 3.921492161 | 12.60892464 |
| chr2:31778333-31784779:+ |  | chr2:31778333-31784779:+ |  | 9.871223444 | 3.921492161 |
| mmu_circ_0009942 |  | chr2:37640850-37647285:- |  | 3.921492161 | 12.97534702 |
| chr2:60001505-60076911:- |  | chr2:60001505-60076911:- |  | 14.40941107 | 3.921492161 |
| chr3:117829576-117846696:- | | chr3:117829576-117846696:- |  | 7.639764159 | 3.921492161 |
| mmu_circ_0001170 |  | chr3:131599864-131607463:+ | | 15.44511641 | 3.921492161 |
| chr3:138955321-138965996:- | | chr3:138955321-138965996:- |  | 11.23529914 | 3.921492161 |
| mmu_circ_0010543 |  | chr3:152420014-152428832:+ | | 3.921492161 | 11.86034007 |
| mmu_circ_0001120 |  | chr3:37448407-37464658:+ |  | 9.55511893 | 3.921492161 |
| chr4:107336113-107341102:+ | | chr4:107336113-107341102:+ | | 3.921492161 | 12.89209258 |
| chr4:130668924-130728164:+ | | chr4:130668924-130728164:+ | | 3.921492161 | 12.7668231 |
| mmu_circ_0011253 |  | chr4:130727997-130730548:+ | | 8.583889358 | 3.921492161 |
| chr4:137948543-137951601:+ | | chr4:137948543-137951601:+ | | 14.96938816 | 3.921492161 |
| mmu_circ_0011389 |  | chr4:143170035-143194734:- |  | 14.30820079 | 3.921492161 |
| mmu_circ_0011582 |  | chr4:35083769-35084389:- |  | 14.01893363 | 3.921492161 |
| mmu_circ_0001204 |  | chr4:48604580-48617331:+ |  | 15.73561714 | 3.921492161 |
| mmu_circ_0011663 |  | chr4:48604580-48650418:+ |  | 15.04211099 | 3.921492161 |
| mmu_circ_0001206 |  | chr4:48636839-48650418:+ |  | 3.921492161 | 13.1857451 |
| mmu_circ_0011689 |  | chr4:55366776-55370472:+ |  | 13.20051254 | 3.921492161 |
| mmu_circ_0011735 |  | chr4:63431849-63435562:- |  | 12.92090216 | 3.921492161 |
| chr4:81303592-81335874:- |  | chr4:81303592-81335874:- |  | 13.28284568 | 3.921492161 |
| chr4:9635900-9639347:- |  | chr4:9635900-9639347:- |  | 12.05447349 | 3.921492161 |
| mmu_circ_0001369 |  | chr5:100805897-100812202:- |  | 3.921492161 | 13.5539977 |
| mmu_circ_0011938 |  | chr5:103783123-103784513:+ | | 3.921492161 | 11.09190923 |
| mmu_circ_0011943 |  | chr5:103885906-103886290:- |  | 3.921492161 | 12.58791449 |
| chr5:108180397-108180891:+ | | chr5:108180397-108180891:+ | | 14.69651162 | 3.921492161 |
| mmu_circ_0001387 |  | chr5:110753803-110756766:- |  | 3.921492161 | 12.97534702 |
| mmu_circ_0001388 |  | chr5:110755396-110756766:- |  | 9.149735463 | 3.921492161 |
| mmu_circ_0001398 |  | chr5:118671012-118680971:+ | | 7.639764159 | 3.921492161 |
| mmu_circ_0001431 |  | chr5:147450652-147455188:+ | | 14.22734297 | 3.921492161 |
| mmu_circ_0012499 |  | chr5:28331110-28352643:+ |  | 3.921492161 | 12.74800535 |
| mmu_circ_0012545 |  | chr5:32819865-32822138:- |  | 14.48091572 | 3.921492161 |
| chr5:5092957-5227258:- |  | chr5:5092957-5227258:- |  | 13.77525061 | 3.921492161 |
| mmu_circ_0012684 |  | chr5:5195277-5249228:- |  | 3.921492161 | 13.36932853 |
| chr5:66307534-66313429:- |  | chr5:66307534-66313429:- |  | 3.921492161 | 7.613111222 |
| mmu_circ_0001352 |  | chr5:66391626-66400281:- |  | 3.921492161 | 13.93292318 |
| mmu_circ_0012878 |  | chr5:96128942-96134132:- |  | 13.81268533 | 3.921492161 |
| chr6:118411954-118418617:- | | chr6:118411954-118418617:- |  | 3.921492161 | 13.05405804 |
| chr6:120862854-120870835:+ | | chr6:120862854-120870835:+ | | 14.2548022 | 3.921492161 |
| mmu_circ_0013120 |  | chr6:134541651-134542045:- |  | 3.921492161 | 13.08437814 |
| chr6:147534028-147537480:+ | | chr6:147534028-147537480:+ | | 3.921492161 | 12.8573973 |
| chr6:149166805-149183538:- | | chr6:149166805-149183538:- |  | 13.75616257 | 3.921492161 |
| chr6:33332210-33347957:+ |  | chr6:33332210-33347957:+ |  | 3.921492161 | 12.70961709 |
| mmu_circ_0001473 |  | chr6:50143718-50163649:+ |  | 3.921492161 | 13.33190819 |
| chr6:51996016-52003755:- |  | chr6:51996016-52003755:- |  | 3.921492161 | 13.87339332 |
| mmu_circ_0013469 |  | chr6:57762434-57790035:- |  | 11.23529914 | 3.921492161 |
| mmu_circ_0001481 |  | chr6:65862914-65901859:+ |  | 3.921492161 | 12.28265039 |
| chr6:84149674-84152409:+ |  | chr6:84149674-84152409:+ |  | 3.921492161 | 12.25625942 |
| chr6:8537816-8582790:+ |  | chr6:8537816-8582790:+ |  | 3.921492161 | 13.19966173 |
| chr6:8537816-8591665:+ |  | chr6:8537816-8591665:+ |  | 13.77525061 | 3.921492161 |
| mmu_circ_0013639 |  | chr6:99003199-99016665:- |  | 14.29503529 | 3.921492161 |
| mmu_circ_0001625 |  | chr7:121052086-121057399:+ | | 14.18514859 | 3.921492161 |
| mmu_circ_0001632 |  | chr7:130218997-130242644:- |  | 14.38476634 | 3.921492161 |
| mmu_circ_0013936 |  | chr7:130504693-130516168:- |  | 3.921492161 | 14.45206849 |
| chr7:4434993-4444321:- |  | chr7:4434993-4444321:- |  | 3.921492161 | 12.97534702 |
| chr7:44983770-44984133:- |  | chr7:44983770-44984133:- |  | 14.15631743 | 3.921492161 |
| mmu_circ_0001571 |  | chr7:56100078-56106532:+ |  | 3.921492161 | 13.02308702 |
| mmu_circ_0001589 |  | chr7:81893798-81905636:- |  | 10.70880892 | 3.921492161 |
| chr7:82428333-82499755:+ |  | chr7:82428333-82499755:+ |  | 3.921492161 | 12.90913226 |
| chr7:82514974-82523333:+ |  | chr7:82514974-82523333:+ |  | 13.45844378 | 3.921492161 |
| mmu_circ_0001591 |  | chr7:84632655-84634409:- |  | 7.639764159 | 3.921492161 |
| mmu_circ_0001601 |  | chr7:99065799-99099473:- |  | 3.921492161 | 11.50444073 |
| mmu_circ_0001605 |  | chr7:99935161-99955540:- |  | 12.85065377 | 14.21229417 |
| mmu_circ_0014548 |  | chr8:109876802-109877083:+ | | 14.30820079 | 3.921492161 |
| mmu_circ_0001662 |  | chr8:18641564-18689059:+ |  | 3.921492161 | 13.81130097 |
| chr8:25088341-25088505:- |  | chr8:25088341-25088505:- |  | 13.20051254 | 3.921492161 |
| mmu_circ_0001669 |  | chr8:36937559-36938758:- |  | 13.61496936 | 3.921492161 |
| mmu_circ_0014982 |  | chr8:77344639-77351672:- |  | 3.921492161 | 12.69003298 |
| mmu_circ_0001692 |  | chr8:77344639-77365160:- |  | 15.41500676 | 3.921492161 |
| mmu_circ_0014994 |  | chr8:78525161-78531158:+ |  | 3.921492161 | 11.75117967 |
| mmu_circ_0001704 |  | chr8:84771783-84772315:- |  | 13.48187143 | 13.38158935 |
| mmu_circ_0015097 |  | chr8:88705225-88719396:+ |  | 14.56009133 | 3.921492161 |
| mmu_circ_0001797 |  | chr9:65794621-65795495:- |  | 13.63600572 | 3.921492161 |
| chr9:7005489-7050483:- |  | chr9:7005489-7050483:- |  | 3.921492161 | 11.54860682 |
| chr9:7031640-7050483:- |  | chr9:7031640-7050483:- |  | 3.921492161 | 12.17406009 |
| chr9:7952952-7973911:- |  | chr9:7952952-7973911:- |  | 7.639764159 | 3.921492161 |
| mmu_circ_0001830 |  | chr9:96602712-96611499:- |  | 3.921492161 | 9.121646537 |
| mmu_circ_0001889 |  | chrX:101213773-101215578:- | | 3.921492161 | 12.90913226 |
| chrX:74241642-74241919:- |  | chrX:74241642-74241919:- |  | 14.90151693 | 3.921492161 |
| chrX:94098282-94102380:- |  | chrX:94098282-94102380:- |  | 12.23075813 | 13.6279074 |
| mmu_circ_0016408 |  | chrX:94098282-94114263:- |  | 14.08161335 | 12.90913226 |

**Table S2 mmu_circ_0008542 sequences.**

>mmu_circ_0008542|ENSMUST00000162336|Rrp15

GAAGCTGTGACTCAGAGATGGACCACTCAGATGATGGAGCTGCAGAAGCAGACAGTGAGGACAATGTTGAATCCTGTGAGGAAGACAATGAAGACGCCGCAGAGTCGAGTGCTGGGACCAATTCAGGCTGGGCAGATGCCATGGCAAAAATCCTTAACAAAAAGACTCCTAAAAGCAAAGCCACCATCCTCACCAAAAACAAAGAGCTGGAGAAGGAGAAGGAGAAGCTAAAGCAGGAGAGGCTGGAGAAAAGGAAGCAGGTGTGTCCGTCCGCCTGCCTGTGCTGTCTCTGCTTAGCAGCAGTCGGCTTTGAGGGCTTGTGAGGCGTGGGTAAGCAGGCTCAACTCAGGAGCGCAGTCTTATGGGTCCACTGAGCAAACCAGCGAGCACTACTGCATAAACCCGTGATACCGGGGGGTGCAGTTCAGGAGTGGGCATTTTCTGTTCATAACTGAATTCTTGCTCTGGTTAGTTGCTCCTGATAGAAGTCAGTCTACTTTAACCTTGATTACAGACCTAAATTTGCATATGAGTTGGCTGTTACATAGAAAAGAAACTATGGTAAGGTTTTGAGGGCCCCTGTACTACAAGTATCACTATGTCAAAAATATTTCCTTATAAAAATAAGTATTTTCTAATGAAAGAAAACTAACCTTTCATATTTGTACTGACAATATAGAAGTAGTAACTTCCAAGGAGTAGTTTCCAGGTACAAATGGTTTAGGTTATACTGCTCTTCATGTCTGGCAGTGCCTACATCACGTCTTTGCTTGCTCTGCTTGAAAGCAGGGCTTGAGGGCCTTGCCCTGAAACACAGCTGTGCTGCTCACACTCAGGATTCTTTTTGTTAAGATTTTTGTCTCTAAACAGAAAGCTTTTCAGCTGAAATGTGAATATGTGTTTATTAACTGGCAAAGGCTACAGCAACCTTTTAAATCTAGATACTAGACTCTACCTAGTCTCCAGATTCTAGGGAGGGAAAGCAGGACAGCACTGAAAGCCAGGGCCAGGCCTGCGGGTCAGCTCCGAGTGTTGGAGCTGAGCTGTGCACAGAGCGCTCAGACCCATCTGCTGACTGTAAAATGTACTGATTTGAGGAAGAGTGCTTTTTCTAAAGGAGCCAGGTGAGGTTGTGCTGTCCAGTCAGGAGCATTCGCCATGTTCCTAGCATACAGTGGAGGAGGGGAGAGAAAAGTTGGCATTTCCCACCATCCTCAGTGTGATGCTTAGCGTCCTTGAGAAGCTCATCTGGCCAGTATGTATCAGCTTGGGAAACACCTCACAGCCATTCTGTGAGATCAGGATGATGCTCAGAGAGCGTTTTACACTAGCCTATGCCTTGTCTGTGAGCCTTTCTTCCTCCAGCTCCCCTTCAGTGTTTGCACCGCGGTAGGAAATCAGTTTACTTGAATACTTGAATTTGTGACTAAAAAGGCTGTGTGTGACAAGAACATTGGTTGTTAGATGTGCCATGAGGGGAGAAATAGGTTTAAATGACATATTAAAGATTTATTTATTTTGTGTGAGTACACTGTGGCTCTCTTCAGACACACCAGAAGAGGGCATCGGATCGCATTACAGATGGTTGTGAGCCACCATGTGGTTGCTGGGAATTGAACTCTGAACCTCTGGAAGAGTTCTTAACTGGAAGTGTTCTTAACTGCTGAGCCATCTCTCCAGCCCTGTTTAAATGGTTTTATATAAAGAGAAATAGATAATACTTGCATATCTGCCTACGATATTGCTCTCTCCAGAGCTGGTACAGACTCTGTCCTGAAGGTTGATGAGTCTGCTGGCCAGGATTGGAAGGCATAGCTTGCCCCTGCTGGGGTAGACAGTGCTGTTGCACATCCCACTGGTGATGCTGGGCAGGTCAGGGGGGTGGCTGCTCACCTTGCTGTGCACTGCCTGGCTGCACATACTGGCAGCTCTCCAGAGCTGATGCCAGAGGATGGACAGCCTCTTAGAGATGCCTCTCAGAAGGCACACAGAGTGCTAACCGCAGGCTCTTTGAGCTGAGCCCTGAAACTGGTTTTAGAAACAGCCTCTGATACCCAGGGGTAGGGTGCTTTATTACCTCCGTCCTTTCACAGATCCTATCAGAGTTTGCTGCACTTCCAAATCACCTAATACCACAGTCATGTAACCATATTCATAATAACCATATTCACCACACAGCTTGATAAGAAGCGGGAGTGGGAAATGCTGTGCAGAGTGAAGCCAGATGTTGTCAAAGACAAAGAGGCAGAGAGGAACCTTCAGAGGATTGCGACAAGGGGTGTGGTGCAGCTCTTCAATGCTGTTCAGAAACACCAAAGGAATGTTGGCGAAAAGGTTAAGGAAGCAGGAGGCTCCGTCCGCAAGCGAGCCAAGCTGATGTCAACTGTTTCCAAGAAGGATTTCATCAGTGTTCTTCGAGGAATGGATGGTACAAGTAGGAACAGTCCTGCTGGGAAGAGCCCCAAAGCCAGACAG

**Table S3. Primer sequences for RT-qPCR.**

| mmu-Ctsk | F: GGTCCCAGACTCCATCG | R: GCTGAAAGCCCAACAGG |
| --- | --- | --- |
| mmu-Mmp9 | F: GACGACATAGACGGCATCC | R: TGGTTCAGTTGTGGTGGTG |
| mmu-Acp5 | F: TTACTACCGTTTGCGCTTC | R: CATTTTGGGCTGCTGACT |
| mmu-ALP | F: ATATGGTAACGGGCCTGGCT | R: TCTTCTCCACCGTGGGTCTC |
| mmu-Bglap | F: CAGTCCCCAGCCCAGAT | R: GCGTTTGTAGGCGGTCTT |
| mmu-Col1a1 | F: CAGAGGCGAAGGCAACA | R: GTCCAAGGGAGCCACATC |
| mmu-Runx2 | F: CATGAGCGGCCACAGAC | R: AGGGCTTTGGGGAGGTT |
| mmu-Tnfrsf11a | F: GACAGGGCTGATGAGAGG | R: CGCTAGAGATGAACGTGGA |
| mmu-Rrp15 | F: TGATGGGAGCCAGCATGAAG | R: ACCGTCGGGTCATTTGCAG |
| mmu-Mettl3 | F: ATGAACGGGTGGATGAAA | R: CCCTGGTTGAATCCTTGA |
| mmu-ALKBH5 | F: GCGTATGGGGCTTAAACA | R: GCAAAAGAGGTCAGAACCA |
| mmu_circ_0008542 Divergent | F: CGAGCCAAGCTGATGTCAAC | R: TCTGAGTCACAGCTTCCTGT |
| mmu_circ_0008542  Convergent | F: TCGAGTGCTGGGACCAATTC | R: TGCTTCCTTTTCTCCAGCCT |
| mmu-GAPDH | F: GTTGCCATCAACGACCCCTT | R: TCCACGACATACTCAGCACC |

**Table S4. RNA oligoribonucleotides.**

| mmu_miR-185-5p | F: TCTGGAGAGAAAGGCAGTTCCTGA | R: mRQ3’ primer |
| --- | --- | --- |
| U6 | F: CTCGCTTCGGCAGCACA | R: AACGCTTCACGAATTTGCGT |
| si-Mettl3 | F: CAAGGAAGAGUGCAUGAAA | R: UUUCAUGCACUCUUCCUUG |
| si-RANK | F: GCAGUAGUCUAAGUGGAAA | R: UUUCCACUUAGACUACUGC |
| mmu_miR-185-5p mimic | UGGAGAGAAAGGCAGUUCCUGA | |
| mmu_miR-185-5p inhibitor | UCAGGAACUGCCUUUCUCUCCA | |
| mmu_circ_0008542-WT | CCTCTGGAAGAGTTCTTAACTGGAAGTGTTCTTAACTGCTGAGCCATCTCTCCAGCCCTGTTTAAATGGT | |
| mmu_circ_0008542-MUT | CCTCTGGAAGAGTTCTTAAGTCCTAGTGTTCTTATGACGGGAGCCAAGAGAGGTGCCCTGTTTAAATGGT | |
| Tnfrsf11a-WT-mmu-miR-185-5p | GAGCTCAAACCCCACACCCAGGACTATCATCATCTCTCCATGCCTACTTCTTTTTTGCTGTACTTCCCTT | |
| Tnfrsf11a-MUT-mmu-miR-185-5p | GAGCTCAAACCCCACACCCAGGACTATCATCAAGAGAGGTTGCCTACTTCTTTTTTGCTGTACTTCCCTT | |

**Table S5. Specific primers for the m6A-modified RNA fragments** **in** **circ_0008542.**

| mmu_circ_0008542-12 | F: CCAAAGCCAGACAGGAAGCT | R: GCTTCTGCAGCTCCATCATC |
| --- | --- | --- |
| mmu_circ_0008542-345 | F: TCAGATGATGGAGCTGCAGA | R: GACTCTGCGGCGTCTTCAT |
| mmu_circ_0008542-6 | F: AATGAAGACGCCGCAGAGTC | R: TGTTAAGGATTTTTGCCATGGC |
| mmu_circ_0008542-7 | F: CCAATTCAGGCTGGGCAGAT | R: TGAGGATGGTGGCTTTGCTT |
| mmu_circ_0008542-8 | F: CCAGATTCTAGGGAGGGAAAGC | R: CTCGGAGCTGACCCGCAG |
| mmu_circ_0008542-9 | F: CACATACTGGCAGCTCTCCA | R: TCTGTGTGCCTTCTGAGAGG |
| mmu_circ_0008542-10 | F: CTGTGCAGAGTGAAGCCAGA | R: CCCTTGTCGCAATCCTCTGA |
