# Supplementary figures and images for: Circ_0008542 in osteoblast exosomes promotes osteoclast-induced bone resorption through m6A methylation
Source: Cell Death Dis. 2021 Jun 18;12(7):628. doi: 10.1038/s41419-021-03915-1 (PMC8213782; doi:10.1038/s41419-021-03915-1)

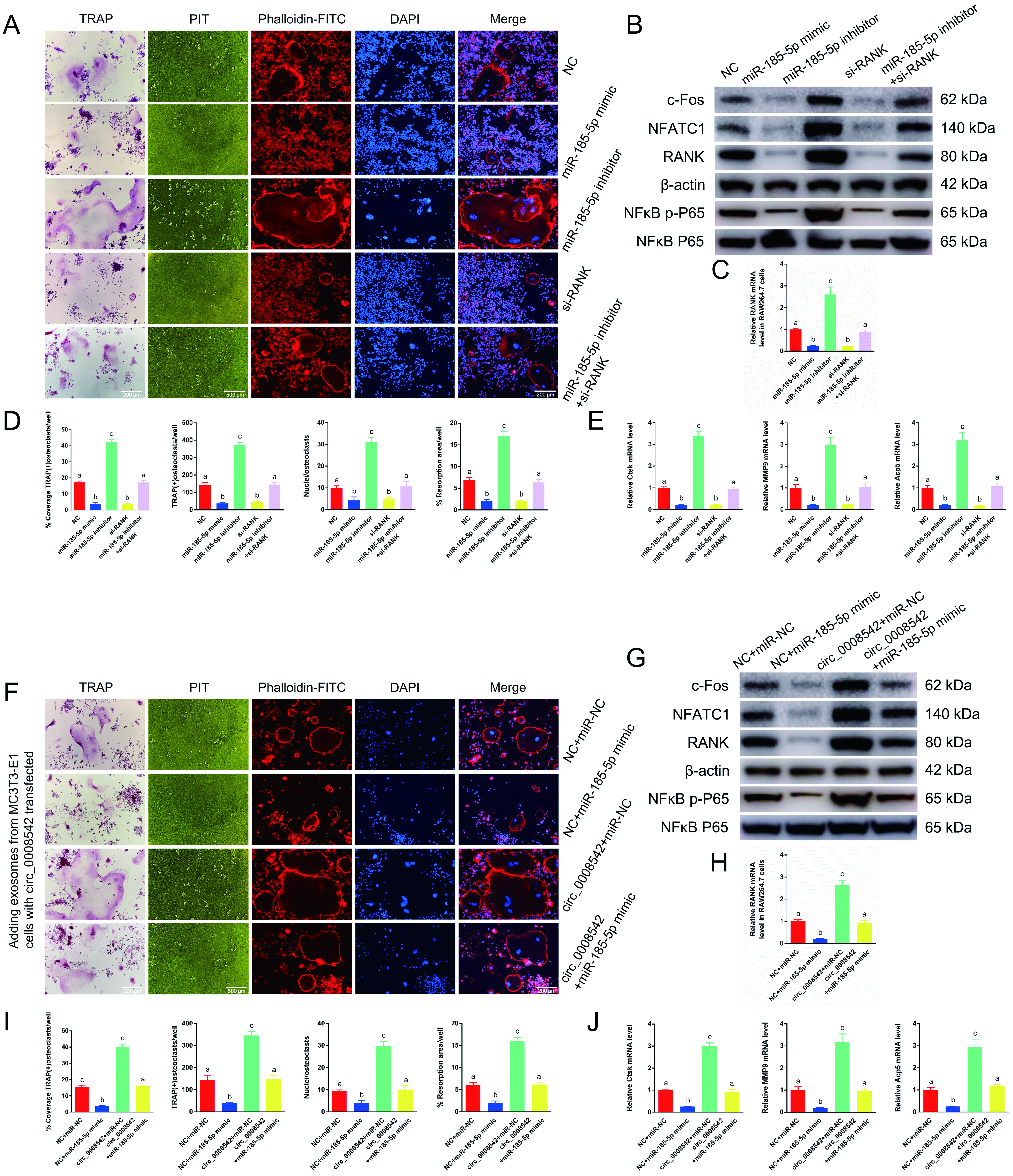

Supplement: Supplementary file 2 — S1 [file 41419_2021_3915_MOESM2_ESM.tif]

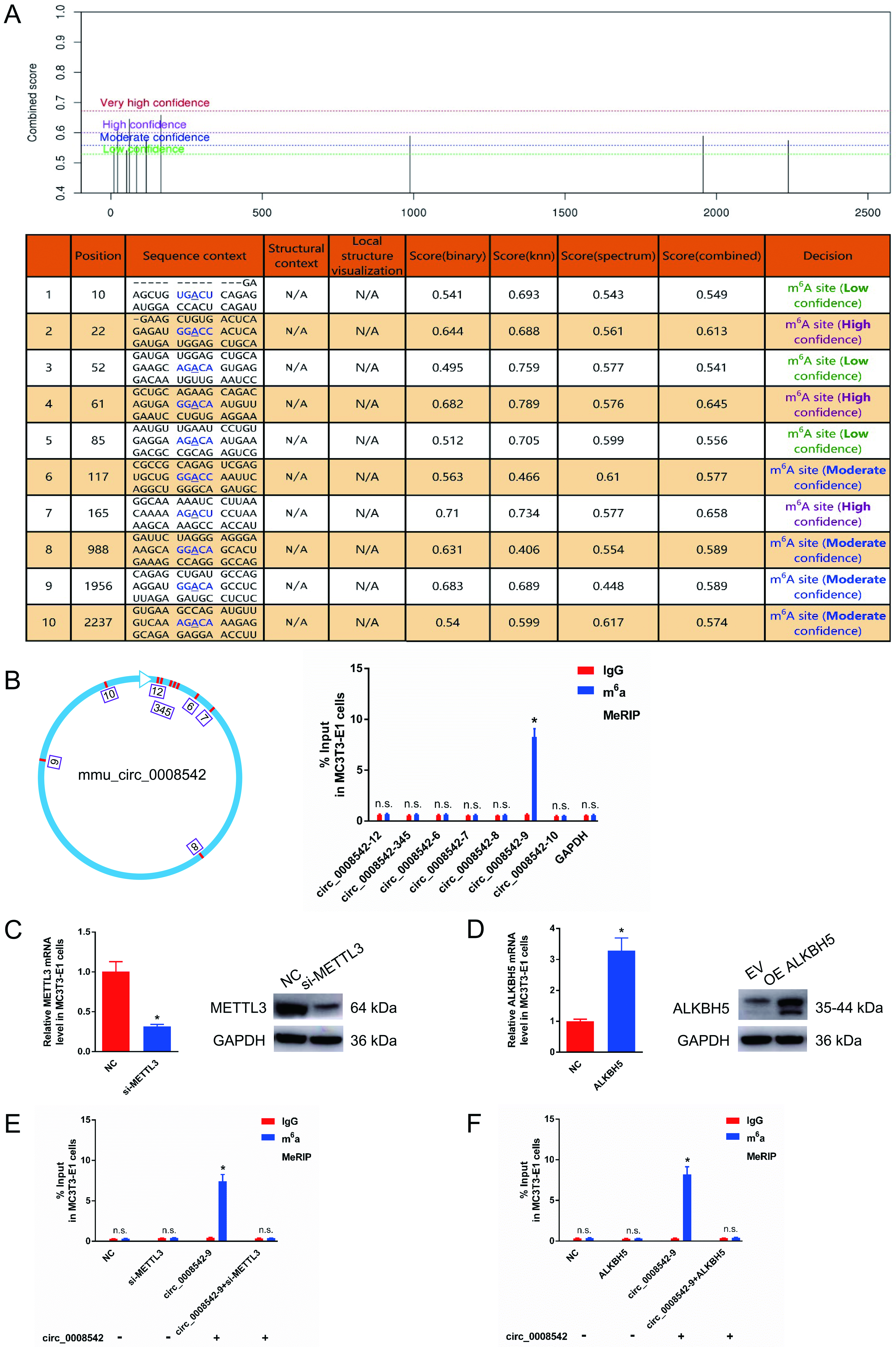

Supplement: Supplementary file 3 — S2 [file 41419_2021_3915_MOESM3_ESM.tif]

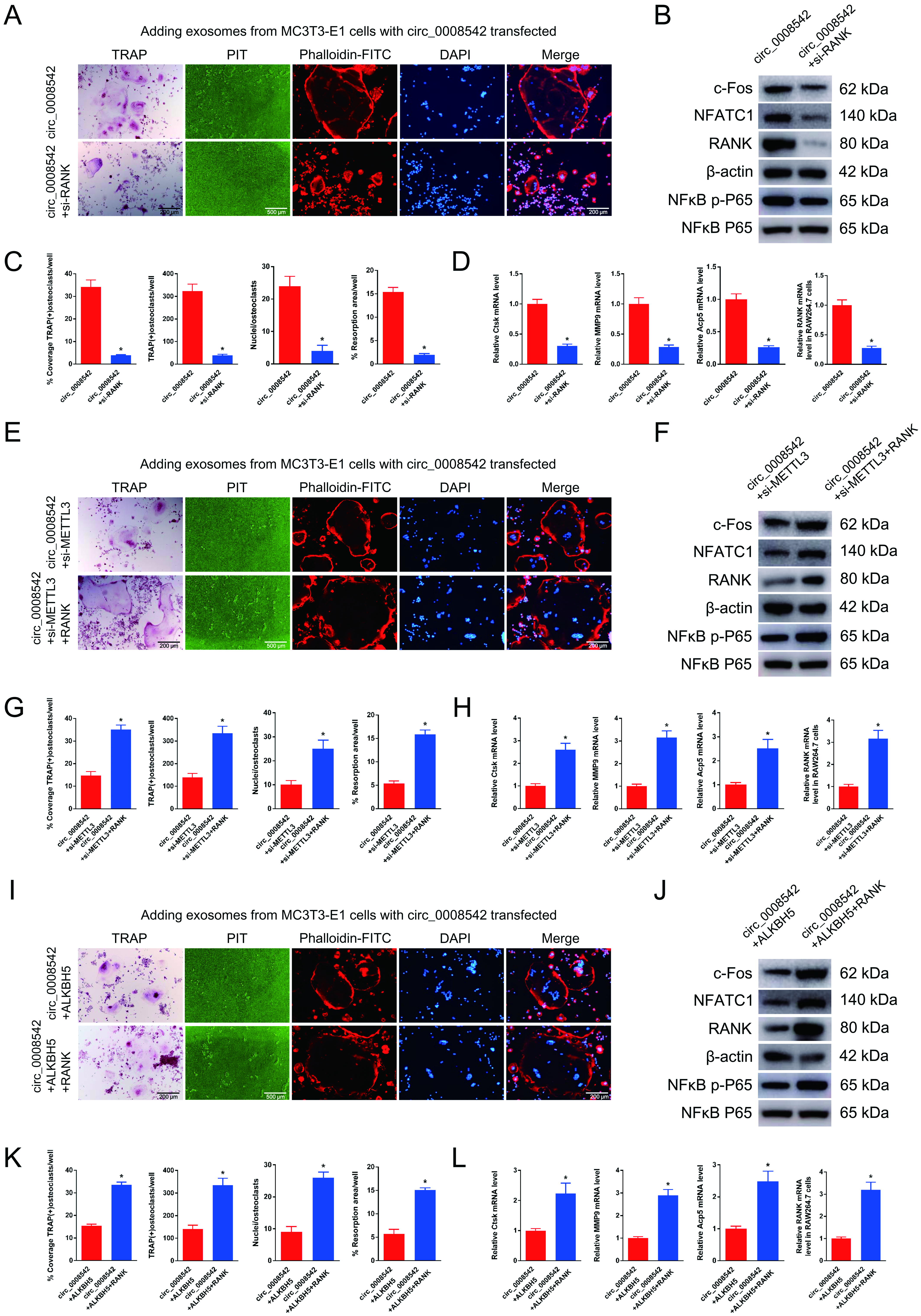

Supplement: Supplementary file 4 — S3 [file 41419_2021_3915_MOESM4_ESM.tif]

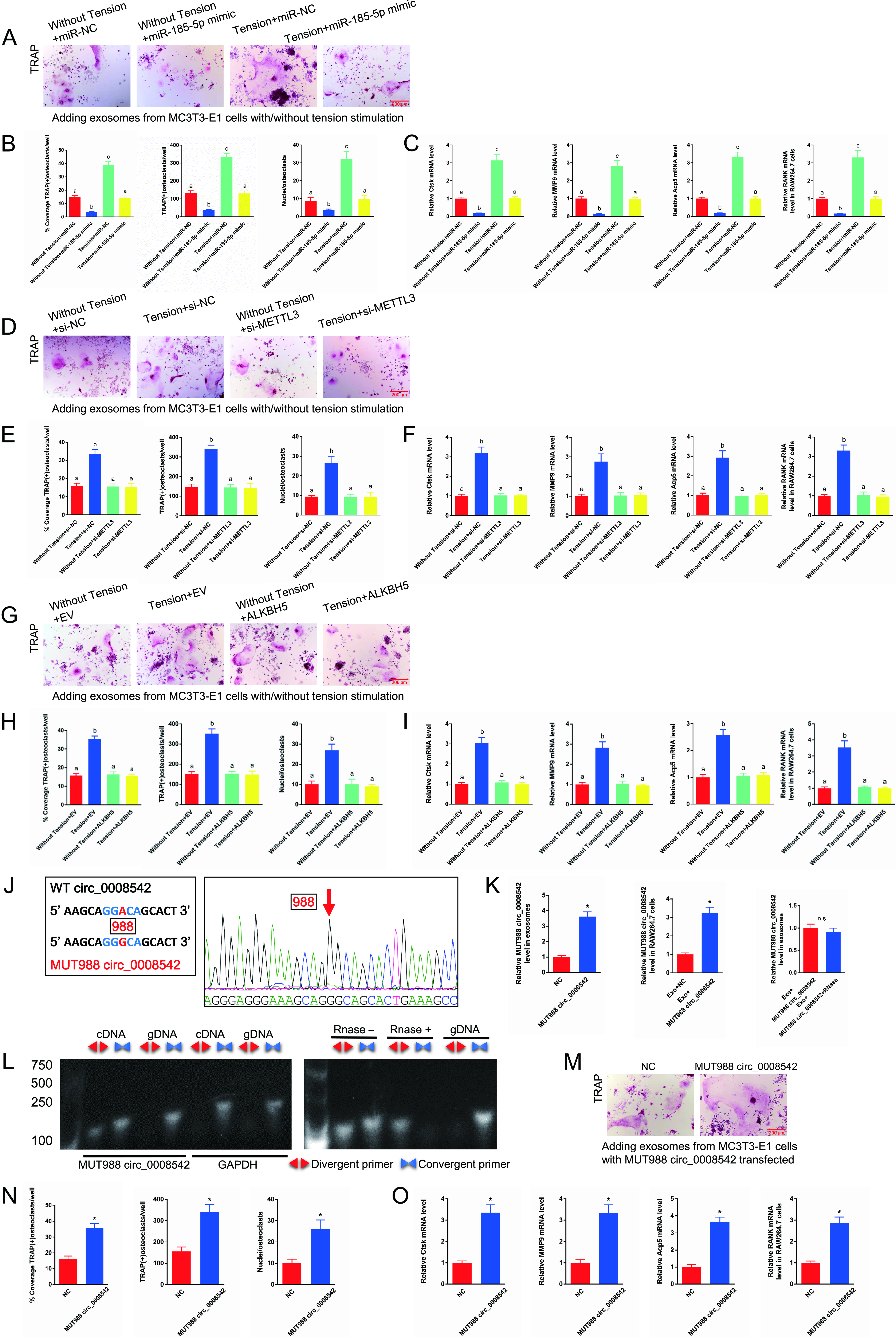

Supplement: Supplementary file 5 — S4 [file 41419_2021_3915_MOESM5_ESM.tif]
